# Synthesis and Characterization of the μ_6_‑F Compounds [NEt_4_][F(Cl_2_)_3_] and [NEt_4_][F(Br_2_)_3_]

**DOI:** 10.1021/acsorginorgau.5c00064

**Published:** 2025-07-24

**Authors:** Jonas R. Schmid, Patrick Pröhm, Patrick Voßnacker, Günther Thiele, Carsten Müller, Sebastian Riedel

**Affiliations:** Freie Universität Berlin, 9166 Institute of Chemistry and Biochemistry, Fabeckstr. 34/36, Berlin 14195, Germany

**Keywords:** bromine, chlorine, fluorine, halogens, X-ray diffraction

## Abstract

The reaction of tetraethylammonium polychlorides or polybromides in propionitrile with substoichiometric amounts of diluted fluorine at low temperatures leads to the non-classical interhalogen compounds [NEt_4_]­[F­(X_2_)_3_] (X = Cl and Br). These compounds are the first examples of anions containing a central μ_6_ fluoride anion, which is octahedrally surrounded by Br_2_ or Cl_2_ units, respectively. Single-crystal X-ray diffraction revealed that these compounds crystallize in the cubic system with every fluoride anion bridged by halogen units, resulting in a 3D network. Furthermore, single-crystal Raman and IR spectra were measured, and investigations using solid-state calculations were carried out.

## Graphical abstract

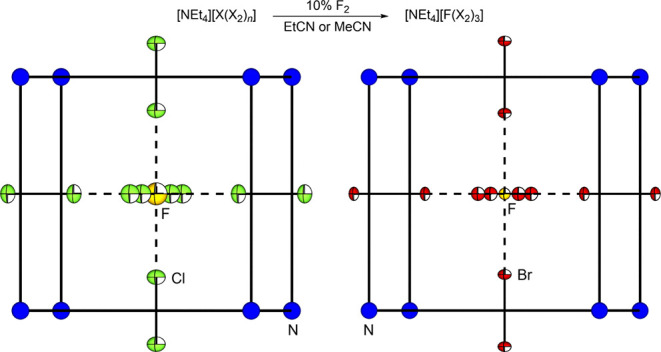

## Introduction

Due to the growing interest in fluorine-halogen compounds and their properties, a wide range of new compounds has been synthesized and structurally described in recent years. They can be divided into classical and non-classical poly­(inter)­halides. Classical interhalides consist of a less electronegative atom surrounded by a more electronegative atoms.^1^ Most fluorine–halogen anions and cations belong to this group, as recently summarized by Kraus et al.^2^ and Riedel et al.^3^ Non-classical interhalide anions and cations have the more electronegative atom surrounded by a less electronegative atoms or molecules.^1^

More recently, some examples of non-classical polyinterhalides with a bridging fluorine atom were reported. Kraus and co-workers first described the μ_2_-F compounds [Br_2_F_5_]^+^, [Br_3_F_8_]^+^ ^4^ and [F­(BrF_3_)_2_]^−^.^5–9^ In addition, following the first observation of a μ_3_-F motive in [F­(IF_5_)_3_]^−^ ^10,11^ a few examples have been reported recently, including the compounds [F­(ClF)_3_]^−^,^3^ [F­(ClF_3_)_3_]^−^,^12^ [F­(BrF_3_)_3_]^−^,^7,13^ [F­(BrF_3_)_3_(BrF_3_)]^−^,^13^ [F­(BrF_5_)_3_]^−^,^2^ [(IF_5_)_6_(HF_2_)_4_]^4–^,^14^ [(BrF_5_)_6_(HF_2_)]^−^ ^15^ and [(BrF_5_)_6_(HF_2_)­(H_2_F_3_)].^2−^ ^15^ A μ_4_-F motive has recently been reported in [F­(BrF_5_)_4_]^−^,^16^ too.

However, non-classical polyinterhalides with a fluorine anion bridging more than four halogen atoms (μ_5_-F and μ_6_-F) are not known thus far. The known compounds are usually obtained by the reaction of the interhalide XF_*n*_ with *n* = 1, 3, 5 with an alkali metal fluoride, [NMe_4_]F or an *in situ* formed fluoride anion. This process is dependent on the stability of the interhalides, which limits the accessible oxidation states in the examples above. By *in situ* oxidation of a halide with diluted fluorine (10–20% in Ar or N_2_) toward the corresponding interhalides, oxidation states like (+I) for bromine are accessible. This synthetic procedure further allows the use of organic cations to stabilize classical and non-classical interhalides of chlorine (+I and +III), bromine (+I and +III), and iodine (+V). These reactions could take place via the oxidation of the halide to the elemental halogen while a fluoride anion is formed (Scheme 1). Additional elemental fluorine can, under certain conditions, lead to a further fluorination of the previously formed halogen. The progress of the reaction can be observed visually by the color change of the solution to the color of the corresponding halogen, followed by a subsequent decolorization (oxidation of the corresponding halogen or intermediate).^9,17^

**Scheme 1:**
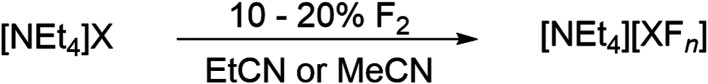
Reaction Pathways toward Classical Interhalides via *In Situ* Oxidation with Diluted Fluorine (X = Cl, Br, with *n* = 2 or 4, or X = I with *n* = 6)

The substoichiometric fluorination of polyhalogens in acetonitrile or propionitrile at low temperatures could enable the *in situ* generation of a fluoride anion that is bridged between halogen units.

## Results and Discussion

Herein, we report the μ_6_-F-bridged compounds [NEt_4_]­[F­(Cl_2_)_3_] and [NEt_4_]­[F­(Br_2_)_3_] with chlorine and bromine in the oxidation state 0. Single crystals suitable for X-ray diffraction were obtained by reacting a solution of tetraethylammonium polychloride or tetraethylammonium polybromide, respectively, with substoichiometric amounts of diluted fluorine (10% in Ar) at −40 °C, followed by cooling slowly to −45 °C or −77 °C, depending on the solvent, shown in Scheme 2.

**Scheme 2:**
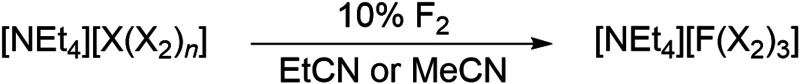
Reaction Pathways toward Non-classical Interhalides via *In Situ* Oxidation of the Halide with Diluted Fluorine (X = Cl, Br)

Single crystal X-ray diffraction analyses revealed that both compounds crystallize in the cubic space group *Pm*3̅*m*. The anionic moiety consists of a central fluoride anion octahedrally coordinated by six halogen units (X_2_), which are bridging between two fluoride anions, resulting in a three-dimensional network. In both structures, the nitrogen positions of the cations and the fluoride position resemble a CsCl structure type. All X–X (X = Cl, Br) and X–F bond lengths are equal due to the cubic crystal system and the corresponding angles are exactly 90° or 180°, see Figure 1. The axes of the unit cell show a lengthening from 711.47(3) pm ([NEt_4_]­[F­(Cl_2_)_3_]) to 753.30(3) pm ([NEt_4_]­[F­(Br_2_)_3_]), which is consistent with the increase in the van der Waals radius of the halogens from chlorine to bromine.^18^

**Figure 1:**
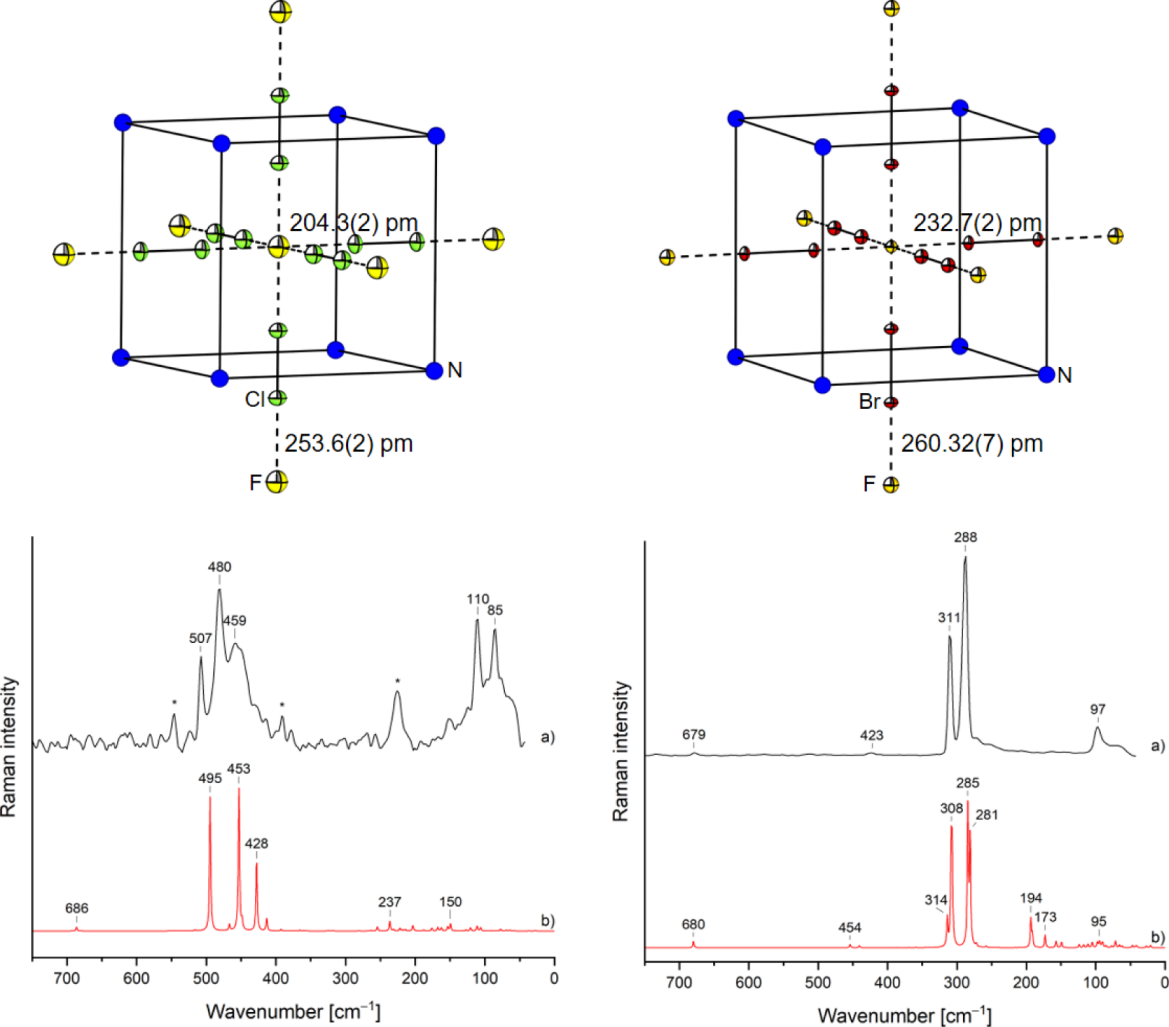
Top: molecular structures in the solid state of [NEt_4_]­[F­(Cl_2_)_3_] (left) and [NEt_4_]­[F­(Br_2_)_3_] (right). Disordered cations are omitted for clarity. Thermal ellipsoids are set to 50% probability for fluorine, chlorine, and bromine. Nitrogen is blue, fluorine is yellow, chlorine is green, and bromine is red. Bottom: comparison of the experimental single crystal Raman spectra of [NEt_4_]­[F­(Cl_2_)_3_] (left) and [NEt_4_]­[F­(Br_2_)_3_] (right) at −196 °C (a) with their corresponding solid-state calculations at B3LYP-D3 (b) level of theory. Bands highlighted with an asterisk correspond to the solvent propionitrile.

When comparing the Cl–F bond length of the bridging fluorine atom in [NEt_4_]­[F­(Cl_2_)_3_] (Cl–F 253.6(2) pm) with those of the non-classical chlorine based polyinterhalides [F­(ClF)_3_]^−^ ^3^ and [F­(ClF_3_)_3_]^−^ ^12^ an elongation up to 34 pm ([F­(ClF)_3_]^−^, *d*_max._(Cl–F) 219.5­(1) pm) or 27 pm ([F­(ClF_3_)_3_]^−^, *d*_max._(Cl–F) 226.5(2) pm) is observed (see Table S1). Contrary to that, the Cl–Cl bond length of [NEt_4_]­[F­(Cl_2_)_3_] (Cl–Cl 204.3(2) pm) is similar to that of the polychloride salts [CCl­(NMe_2_)_2_]­[Cl­(Cl_2_)_3_] (204.0(1) pm, 204.5(1) pm, 204.9(1) pm) and [Cp*_2_Fe]_2_[Cl_20_] (203.6(1) pm) which also contains bridging chlorine units.^19^ In contrast, the octahedral μ_6_-Cl polychloride in [PNP]­[Cl_13_] possesses marginally shorter Cl–Cl bonds (202.2(1) pm, 201.8(1) pm, 201.7(1) pm),^20^ which represents the largest chlorine-based mono anion. Since the μ_6_-F bond lengths are rather long compared to the μ_3_-F reference systems known in literature and the Cl–Cl bond lengths are close to the bond length of elemental chlorine (198.5(2) pm),^21^ the halogen bond should be rather weak. This is evident from the fact that the compound is only stable in the presence of chlorine at low temperatures and already decomposes in an argon stream at −70 °C with a loss of chlorine. The stability of non-classical interhalides and polyhalides was further investigated by quantum-chemical calculations and their results are in good agreement with the observed stability of [NEt_4_]­[F­(Cl_2_)_3_] (see Table S3).

The solid-state structure of [NEt_4_]­[F­(Br_2_)_3_] possesses an octahedrally coordinated fluoride anion (μ_6_-F), analogous to the structure of [NEt_4_]­[F­(Cl_2_)_3_], which bridges between the Br_2_ units. Comparing the Br–F bond lengths in non-classical bromine-containing (+III and +V) polyinterhalide anions and cations toward [NEt_4_]­[F­(Br_2_)_3_] (Br–F 260.32(7) pm, Br–Br 232.7(2) pm) a similar trend can be observed. The Br–F bond lengths in [NEt_4_]­[F­(Br_2_)_3_] are significantly longer than in all μ_2_-F and μ_3_-F BrF_3_-based non-classical polyinterhalides. A similar trend is observed for the bond lengths in μ_3_-F and μ_4_-F BrF_5_-based non-classical polyinterhalides, which are between 14 and 9 pm (see Table S1) shorter than the bond length in [NEt_4_]­[F­(Br_2_)_3_]. Surprisingly, the bromine intercalation compounds CsF·Br_2_ (Br–F 252.1 pm, Br–Br 232.4 pm)^22^ (see Figure S7) and 2CsF·Br_2_ (Br–F 250 pm, Br–Br 233(2) pm)^23^ (see Figure S8) show similar Br–F and Br–Br bond lengths as [NEt_4_]­[F­(Br_2_)_3_]. The three above-mentioned molecular structures in the solid state contain a six-fold coordinated fluoride anion. The fluoride anion in CsF·Br_2_ is coordinated in a quadratic bipyramidal fashion by four cesium atoms in the equatorial position and by two bromine units in the axial position, which form a bridge to the next fluoride anions, resulting in a chain-like structure. In contrast, the fluoride anion in 2CsF·Br_2_ is coordinated in a distorted octahedral fashion toward five cesium atoms and one bridging bromine unit, without the formation of a chain-like structure.

The bromine units in the above-mentioned systems (e.g., Br–Br 232.7(2) pm in [NEt_4_]­[F­(Br_2_)_3_]) are only slightly elongated compared to elemental bromine (229.4(2) pm)^21^ and comparable to [PNP]­[Br_11_·Br_2_] (233.31(3) pm, 232.02(3) pm, 233.52(3) pm, 232.76(3) pm, 233.02(3) pm), which also contains multiple bridging Br_2_ units.^24^ This again indicates that the halogen bond is relatively weak, which is in good agreement with our quantum-chemical calculations (see Tables S3 and S4) and the trend for the [X­(X_2_)_*n*_]^−^ bond lengths in classical and non-classical interhalogen compounds (visualized in Section S4.3).

### Raman Spectroscopy

The experimental single-crystal Raman spectrum of [NEt_4_]­[F­(Cl_2_)_3_] at −196 °C shows three bands at 507 cm^–1^ (A_1g_), 480 cm^–1^ (E_g_), and 459 cm^–1^ (E_g_), which can be assigned to the E_g_ and A_1g_ modes under the assumption of a local octahedral symmetry. This assignment is in good agreement with periodic solid-state calculations using the Crystal program and the B3LYP-D3 DFT functional (see Figure 1; for full spectrum, see Figure S1, and for computational details, see Section S4.8). The two additional bands at 110 cm^–1^ and 85 cm^–1^ can be assigned to lattice mode frequencies of solid chlorine.^25^

The single-crystal Raman spectrum of [NEt_4_]­[F­(Br_2_)_3_] at −196 °C shows two out of three bands at 311 cm^–1^ (A_1g_) and 288 cm^–1^ (E_g_), which can be assigned to the E_g_ and A_1g_ modes under the assumption of a local octahedral symmetry. The third band is not visible, as two bands overlap. The additional band at 679 cm^–1^ can be assigned to ν­(CN) of the cation. The assignment is in good agreement with the above-mentioned periodic solid-state calculations (see Figure 1; for full spectrum see, Figure S3, and for computational details, see Section S4.8.

### Bonding in Non-classical Polyinterhalides

The bonding situation in non-classical poly­(inter)­halides is based on the concept of halogen bonding.^1^ This can be described as a net attractive interaction between an electrophilic region (σ-hole) associated with a halogen atom in one molecule and a nucleophilic region in another or the same molecule in analogy to hydrogen bonding. The halogen bond is more directional than the hydrogen bond and the R–X···Y angle tends to be close to 180° (R–X is the halogen bond donor and Y is the halogen bond acceptor), which is observed in the solid-state structures of [NEt_4_]­[F­(Cl_2_)_3_] and [NEt_4_]­[F­(Br_2_)_3_]. According to the IUPAC definition, the donor of the halogen bond accepts the electron density (Lewis acid, X_2_ or XF_*n*_, X = Cl, Br, I) from the acceptor of the halogen bond (Lewis base X^–^ with X = F, Cl, Br, I, or amines).^26^ This concept can be used for the above-mentioned non-classical polyinterhalide anions and cations, as well as amine halogen or interhalogen compounds such as pyridine·BrF_3_.^27^ The σ-hole can be visualized by mapping the electrostatic potential onto isosurfaces of their electron densities as shown for the halogens, interhalides XF_3_, XF_5_ (X = Cl, Br, I), as well as [F­(Cl_2_)_3_]^−^ and [F­(Br_2_)_3_]^−^ in Figures S10–S13.

### Quantum Chemistry

The non-classical interhalides [F­(X_2_)_*n*_]^−^ (X = Cl, Br, I, *n* = 1–6) were investigated toward their fluoride halogen interaction strength and compared to the corresponding polyhalides [X­(X_2_)_*n*_]^−^ (X = Cl, Br, I) at B3LYP-D4/def2-QZVPPD and PBE0-D4/def2-QZVPPD level of theory. In all cases, the largest stable polyhalide and non-classical interhalide would be [X_7_]^−^ or [F­(X_2_)_3_]^−^ (X = Cl, Br, I) based on the Gibbs free energies (298.15 K, 1.00 atm) of the above-mentioned halide halogen interactions (Tables S3 and S4). For non-classical interhalides of the type [F­(X_2_)_*n*_]^−^ (X = Cl, Br, I, *n* = 1–6), the bond strength increases from X = Cl to X = I, and the lower vapor pressure of the heavier halogens could facilitate their synthesis. However, the increased chemical reactivity of the heavier halogens may result in their oxidation, as demonstrated by Seppelt and co-workers for the CsF/I_2_ system.^23^ The calculated energies for the stability of the non-classical interhalides align well with the experimentally observed stability of [NEt_4_]­[F­(Cl_2_)_3_] and [NEt_4_]­[F­(Br_2_)_3_]. Larger non-classical interhalides may be synthetically accessible, even though they are unstable based on their Gibbs free energies, which were determined by quantum-chemical calculations for the isolated anions. Polyhalides such as [Cl_13_]^−^ are also unstable based on their calculated Gibbs free energies but show additional halogen bonds in the crystal structure, as well as other energetically favorable interactions in the solid state that further stabilize these compounds. Furthermore, they are often prepared in an excess of the corresponding halogen and/or at low temperatures to further stabilize the target compound.^1^

Additionally, the density of states (DOS) for [NEt_4_]­[F­(Cl_2_)_3_] and [NEt_4_]­[F­(Br_2_)_3_] was investigated using periodic solid-state calculations (see Section S4.8), and compared to the results from Macchi, Casati, and coworkers.^28^ Based on our calculations, the systems seem to be semiconductors with band gaps below 3.0 eV, similar to that of [NEt_4_]­[I_3_·(I_2_)_2_]. However, for [NEt_4_]­[F­(Cl_2_)_3_], the compression of the unit cell volume to a similar degree as applied by Macchi, Casati, and co-workers to [NEt_4_]­[I_3_·(I_2_)_2_] increased the band gap, instead of reducing it as in [NEt_4_]­[I_3_·(I_2_)_2_].

## Conclusion

In conclusion, the non-classical interhalides [NEt_4_]­[F­(Cl_2_)_3_] and [NEt_4_]­[F­(Br_2_)_3_] could be obtained by substoichiometric fluorination of the corresponding polyhalides in acetonitrile or propionitrile at low temperatures. These are the first examples of anions containing a central μ_6_-fluorine atom surrounded by halogen atoms in an octahedral fashion. The compounds were analyzed using Raman and IR spectroscopy, and single-crystal X-ray diffraction. The results are supported by quantum chemical calculations in both the gas phase and the solid state.

## Experimental Section

**Caution!** Fluorine, even under dilute conditions, is extraordinarily reactive and can react violently with organic materials under the formation of HF. All reactions should be conducted under rigorously anhydrous conditions.

### Materials, Chemicals and Procedures

All water and air-sensitive substances were handled under argon (Ar 5.0) atmosphere using standard Schlenk techniques and a vacuum up to 10^–3^ mbar. If not mentioned otherwise, all syntheses were performed under stated conditions. All glassware was flame-dried and joints were sealed with Triboflon grease. Single crystals were picked at −80 °C under a nitrogen atmosphere and mounted on a Mitegen micromount using perfluoroether oil diluted with perfluorohexane. Crystal data were collected on a Bruker D8 Venture diffractometer equipped with a Photon II area detector with MoK_α_ radiation at 100(2) K. Multiscan absorption correction was applied as implemented in APEX IV (SADABS-2016/2).^29,30^ The structures were solved with the ShelXT^31^ structure solution program using intrinsic phasing and refined with the ShelXL^32^ refinement package employing least-squares minimizations by using OLEX2.^33^ For visualization, the Diamond V4.65 program package was used.^34^ Raman spectra were recorded at −196 °C on a Bruker MultiRAM II and Ramanscope equipped with a low-temperature Ge detector (1064 nm, up to 450 mW, resolution 4 cm^–1^). IR spectra were recorded on a Nicolet iS50 Advance FTIR by Thermo Fisher Scientific equipped with an ATR unit, with a Ge on KBr beamsplitter and a DLaTGS-KBr detector for MIR and a solid-substrate beamsplitter with a DLaTGS-PE detector for FIR. For low-temperature measurements, a metal cylinder cooled by a cold N_2_ stream was used.^35^

Raman and IR spectra were processed using OPUS 7.5 ^36^ and OMNIC 9.7.46;^37^ Origin Pro 2022 ^38^ was used for their graphical representation.

Quantum chemical calculations were conducted using the HPC system provided by Freie Universität’s IT-service FUB-IT.^39^ The programs Orca 5.0.3, Orca 6.0.1,^40–42^ and Gaussian 16 ^43^ were used in combination with the B3LYP-D4,^44–46^ B3LYP-D3­(*BJ*),^44,45,47,48^ PBE0-D4,^46,49^ M062X,^50^ DFT functionals and def2-QZVPP and def2-QZVPPD basis set.^51,52^ Solid-state calculations were conducted with the B3LYP-D3 ^44,45,47^ functional as implemented in Crystal17 ^53^ and special basis sets, as stated in Section S4.8 were used.^54–57^ Electronic potentials mapped onto electron densities were visualized with VMD 1.9.3 ^58^ and Multiwfn 3.8 was additionally used for calculating ADCH charges.^59–62^

#### [NEt_4_]­[F­(Cl_2_)_3_]

A glass Schlenk tube was charged with [NEt_4_]Cl (200 mg, 1.21 mmol, 1.00 equiv) and saturated with elemental chlorine (5.81 mmol, 4.80 equiv) by condensation onto the salt at −196 °C. The mixture was allowed to reach r.t., excess chlorine was removed via the Schlenk line and the polychloride dissolved in propionitrile (3 mL). Additional chlorine (8.14 mmol, 6.72 equiv) was condensed onto the reaction mixture and the mixture was warmed to −45 °C. Dilute fluorine (10% in Ar, 20 mL min^–1^, 7 min, 0.52 equiv) was bubbled through the reaction mixture. Subsequently, Ar (20 mL min^–1^, 4 min) was passed through the reaction mixture to get rid of any excess fluorine gas. The reaction mixture was slowly cooled to −77 °C to obtain single crystals suitable for X-ray diffraction. The yield could not be determined, as decomposition occurred during attempted isolation of the compound.

Raman (−196 °C, 512 scans): ν̃ = 507 cm^–1^ (m), 480 cm^–1^ (s), 459 cm^–1^ (m).

#### [NEt_4_]­[F­(Br_2_)_3_]

A glass Schlenk tube was charged with [NEt_4_]Br (200 mg, 0.96 mmol, 1.00 equiv) and bromine (290 mg, 1.84 mmol, 1.92 equiv) was condensed onto the salt at −196 °C. The mixture was allowed to reach r.t. and dissolved in propionitrile (4 mL) and cooled to −40 °C. Dilute fluorine (10% in Ar, 20 mL min^–1^, 7 min, 0.65 equiv) was bubbled through the reaction mixture. Subsequently, Ar (20 mL min^–1^, 4 min) was passed through the reaction mixture to get rid of any excess fluorine gas. The reaction mixture was slowly cooled to −77 °C to obtain single crystals suitable for X-ray diffraction. The same reaction conditions can be used with acetonitrile as the solvent, however, crystallization occurs at −45 °C. The yield could not be determined, as decomposition occurred during attempted isolation of the compound.

Raman (−196 °C, 256 scans): ν̃ = 3082 cm^–1^ (w), 2989 cm^–1^ (w), 2939 cm^–1^ (w), 1518 cm^–1^ (w), 311 cm^–1^ (s), 288 cm^–1^ (s), 97 cm^–1^ (w).

IR (ATR, −70 °C, 32 scans): ν̃ = 2975 cm^–1^ (m), 1478 cm^–1^ (s), 1448 cm^–1^ (m), 1433 cm^–1^ (s), 1390 cm^–1^ (m), 1363 cm^–1^ (s), 1170 cm^–1^ (s), 1502 cm^–1^ (m), 997 cm^–1^ (s), 782 cm^–1^ (s), 574 cm^–1^ (w), 419 cm^–1^ (w), 159 cm^–1^ (w), 63 cm^–1^ (s).

## Supplementary Material





## Data Availability

The data underlying this study are available in the published article and its Supporting Information.

## Notes

IR and Raman spectra of [NEt_4_]­[F­(Cl_2_)_3_] and [NEt_4_]­[F­(Cl_2_)_3_], crystallographic information, computational details for polyhalides and non-classical interhalide, their representation and Cartesian coordinates and additional reference^63^ (PDF)
Additionally, tabulated energy values from quantum-chemical calculations (XLSX)
