# Supplementary material for: Synthesis and Characterization of the μ6‑F Compounds [NEt4][F(Cl2)3] and [NEt4][F(Br2)3]
Source: ACS Org Inorg Au. 2025 Jul 24;5(5):400–5. doi: 10.1021/acsorginorgau.5c00064 (PMC12492038; doi:10.1021/acsorginorgau.5c00064)
Supplement: Supplementary file 1 [file gg5c00064_si_001.pdf]

Supporting Information

Synthesis and characterisation of the μ_6-F compounds $[NEt_4][F(Cl_2)_3]$ and $[NEt_4][F(Br_2)_3]$.

Jonas R. Schmid,[a] Patrick Pröhm,[a] Patrick Voßnacker,[a] Günther Thiele,[a] Carsten Müller,[a] Sebastian Riedel[a]*

[a] Freie Universität Berlin, Institute of Chemistry and Biochemistry

Fabeckstr. 34/36, 14195 Berlin, Germany

E-mail: s.riedel@fu-berlin.de

Table of content:

1. Vibrational spectroscopy: 3

2. Non-classical fluorine bridged interhalides and halogen cations: 6

2.1 Depiction of the solid-state structure of CsF·Br_2 and 2CsF·Br_2: 6

3. Crystallographic information: 7

4. Quantum-chemical calculations: 8

4.1 Electrostatic potential of halogens, fluorine containing interhalides, $[F(Cl_2)_3]^-$ and $[F(Br_2)_3]^-$: 8

4.2 Energy calculations: 10

4.3 Quantum-chemically optimized structures: 11

4.4 XYZ files optimized with B3LYP-D4/def2-QZVPPD (Orca 6.0.1): 14

4.5 XYZ files optimized with PBE0-D4/def2-QZVPPD (Orca 6.0.1): 28

4.6 XYZ files optimized with M062X/def2-QZVPPD (Orca 6.0.1): 43

4.7 XYZ files optimized with B3LYP-D3(*BJ*)/def2-QZVPPD (Orca 5.0.3): 47

4.8 Solid-state calculations with the Crystal program: 50

4.8.1 Calculated density of states: 51

5. Literature: 53

1. Vibrational spectroscopy:

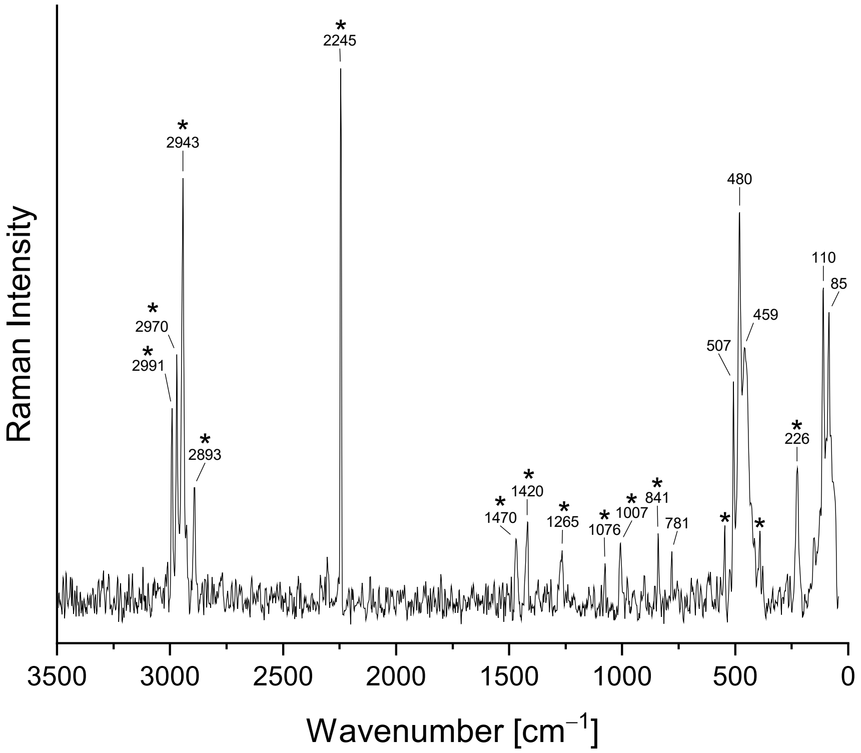

Figure S1: Full single crystal Raman spectrum of $[NEt_4][F(Cl_2)_3]$ at −196 °C. Bands highlighted with an asterisk correspond to the solvent EtCN.

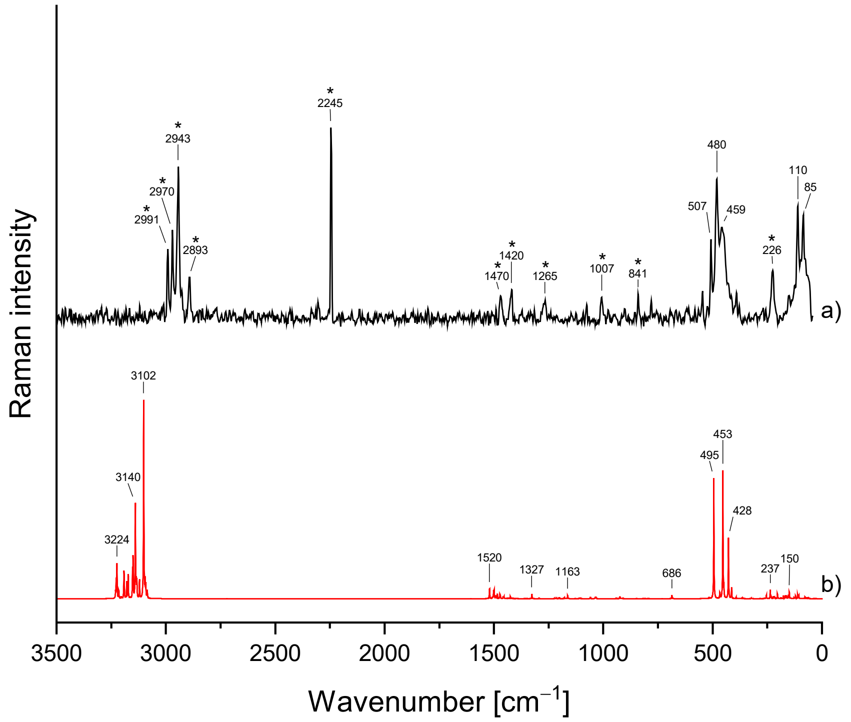

Figure S2: Comparison of the single crystal Raman spectrum of $[NEt_4][F(Cl_2)_3]$ at –196 °C a) with solid-state calculations at B3LYP level of theory b). Bands highlighted with an asterisk correspond to the solvent propionitrile.

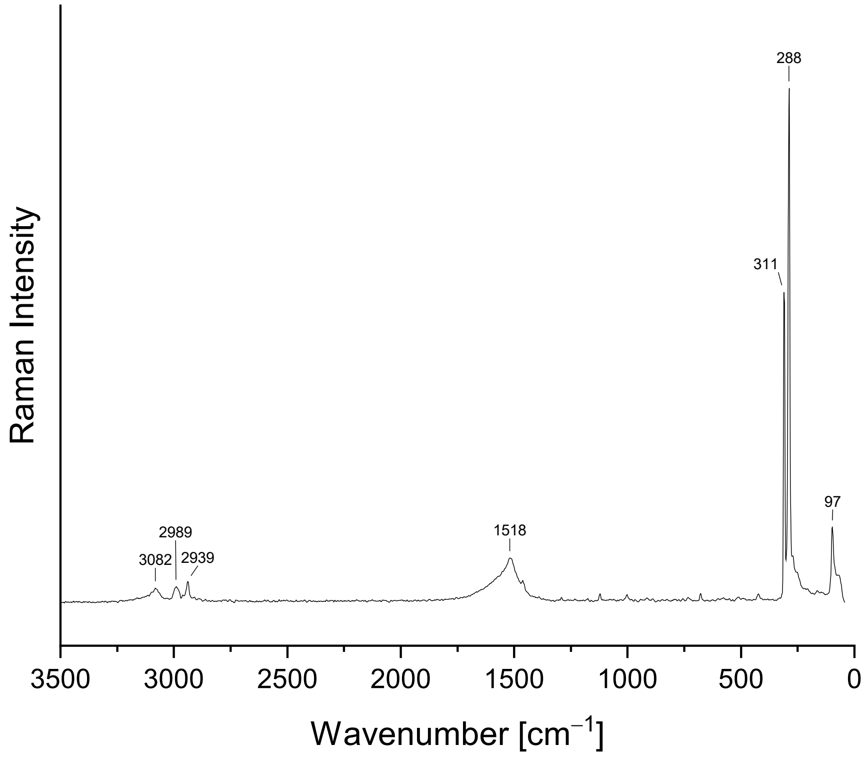

Figure S3: Full single crystal Raman spectrum of [NEt$_4$][F(Br$_2$)$_3$] at −196 °C.

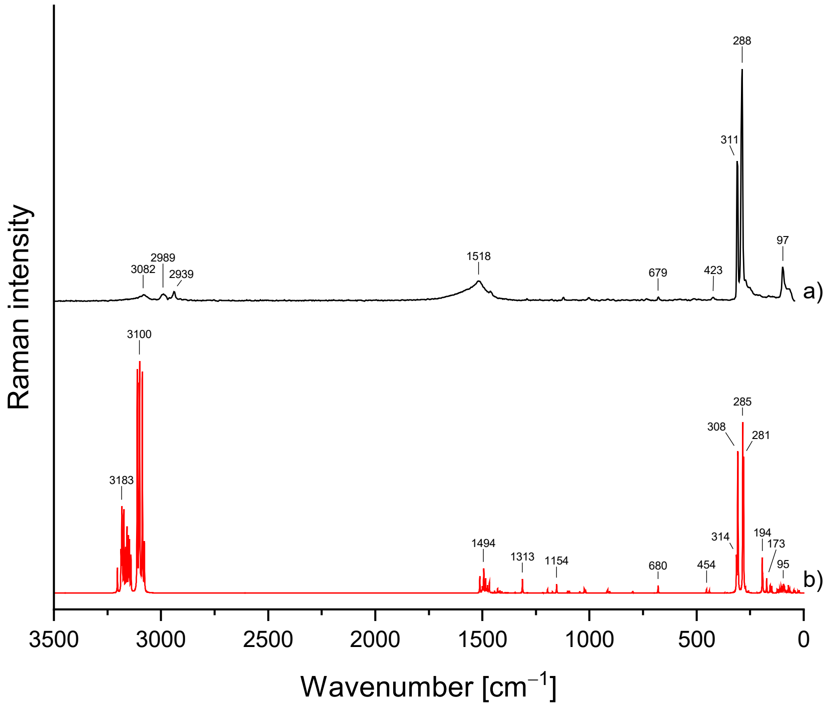

Figure S4: Comparison of the single crystal Raman spectrum of [NEt$_4$][F(Br$_2$)$_3$] at –196 °C a) and the solid-state calculations at B3LYP level of theory b).

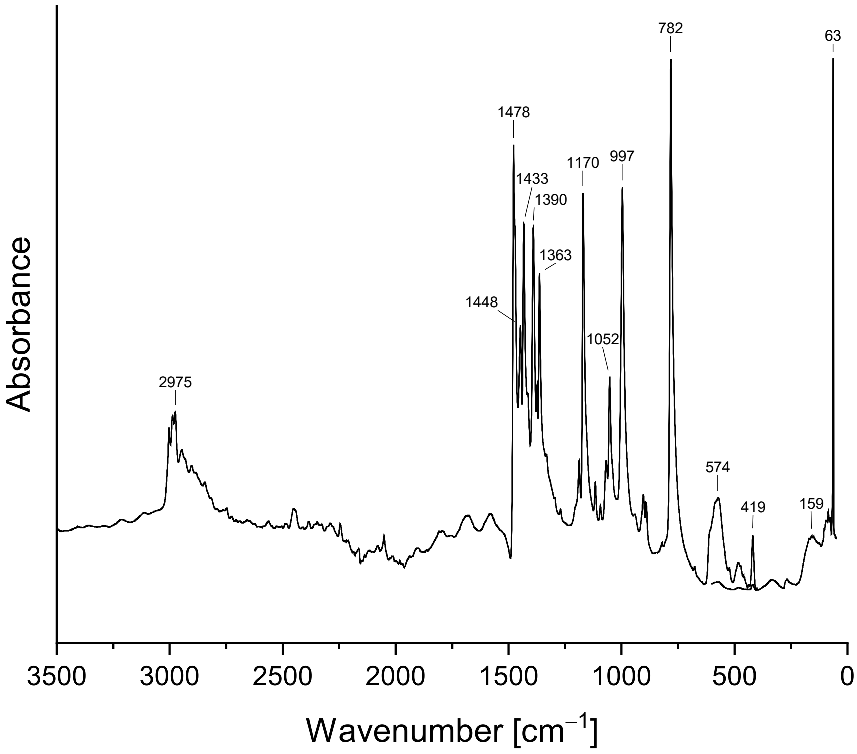

Figure S5: Full IR spectrum of [NEt4][F(Br2)3] (MIR from 3500 to 400 cm^{-1} and FIR from 600 to 50 cm^{-1} overlaid) at −70 °C.

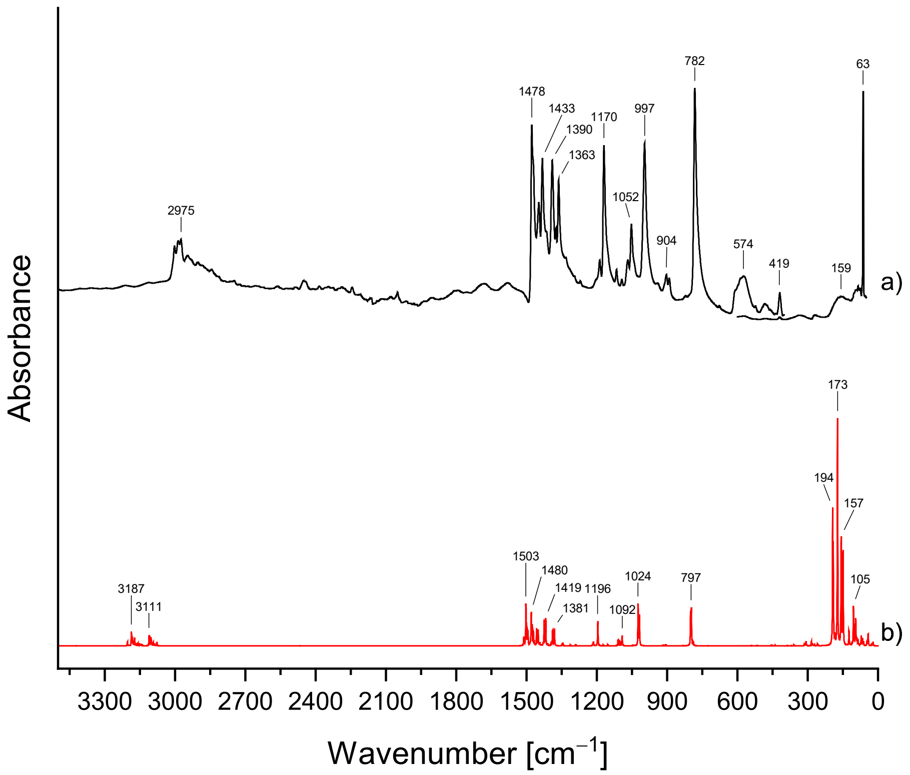

Figure S6: Comparison of IR spectrum of [NEt4][F(Br2)3] (MIR from 3500 to 400 cm^{-1} and FIR from 600 to 50 cm^{-1} overlaid) at −70 °C a) and the solid-state calculations at B3LYP level of theory b).

2. Non-classical fluorine bridged interhalides and halogen cations:

Table S1: Bond lengths in fluorine anion bridged non-classical interhalides and halogen cations.

X	μ_γ-F	Compound	Ox. state of X	μ_γ-F-X bond length in pm	Literature
**Cl**	μ_3-F	$[NMe_4][F(ClF)_3]$	+1	219.4(2) pm, 219.5(1) pm, 213.9(2) pm	3
		$Cs[F(ClF_3)_3]$	+3	224.3(2) pm, 226.3(2) pm, 226.5(2) pm	12
**Br**	μ_2-F	$[Br_2F_5][SbF_6]$	+3	204.1(3) pm, 204.2(3) pm	4
		$[Br_3F_8][SbF_6]$	+3	196.9(6) pm to 217.7(7) pm	4
		$Rb[F(BrF_3)_2]$	+3	211.5(2) pm, 214.5(2) pm	7
		$Cs[F(BrF_3)_2]$	+3	211.3(1) pm, 214.3(1) pm	5,6
		$[PbF][F(BrF_3)_2]$	+3	220(1) pm, 225(1) pm	8
		$[NEt_3Me][F(BrF_3)_2]$	+3	206.68(7) pm, 218.61(7) pm	9
	μ_3-F	$Rb[F(BrF_3)_3]$	+3	224.3(3) pm, 224.8(2) pm, 232.0(3) pm	7
		$Cs[F(BrF_3)_3$	+3	224(1) pm, 224.6(9) pm, 233(1) pm	7
		$Ba[F(BrF_3)_3]_2\cdot BrF_3$	+3	230.93(6) pm	13
		$Ba_2[F(BrF_3)_3]_2[F(BrF_3)_3(BrF_3)]_2$	+3	224.3(6) pm to 233.9(6) pm	13
		$Cs[F(BrF_5)_3]$	+5	246.2(2) pm	2
	μ_4-F	$[NMe_4][F(BrF_5)_4]\cdot BrF_5$	+5	247.4(3) pm, 254.5(2) pm	16
**I**	μ_2-F	$[NMe_4][F(IF_5)]$	+5	282(2) pm, 211(2) pm	63
	μ_3-F	$K[F(IF_5)_3]$	+5	248(1) pm to 251(1) pm	10
		$Cs[F(IF_5)_3]$	+5	252.4(7) pm	10

2.1 Depiction of the solid-state structure of $CsF\cdot Br_2$ and $2CsF\cdot Br_2$:

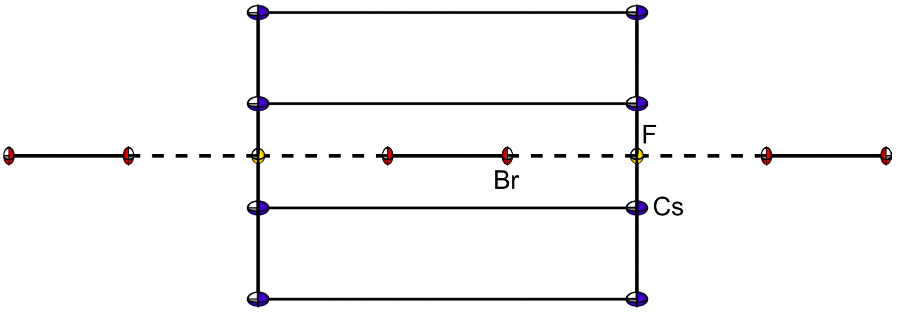

Figure S7: Graphical representation of the unit cell of $CsF\cdot Br_2$. Cesium is blue, fluorine is yellow, and bromine is red.[22]

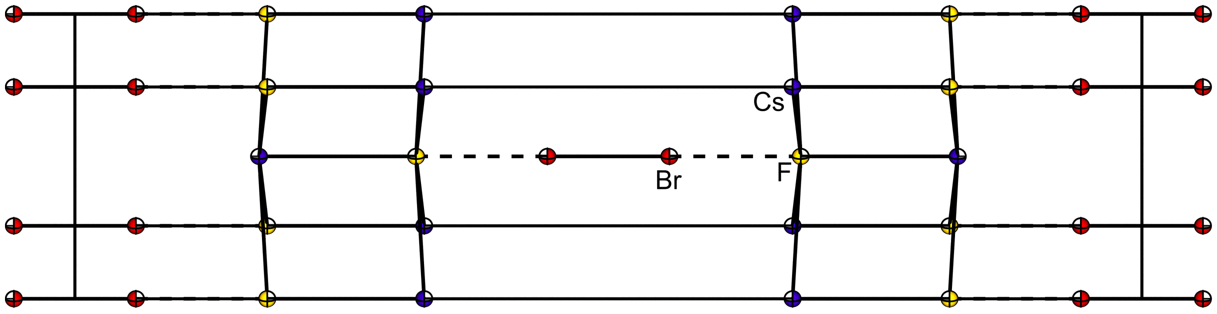

Figure S8: Graphical representation of the unit cell of $CsF\cdot Br_2$. Cesium is blue, fluorine is yellow, and bromine is red.[23]

3. Crystallographic information:

Table S2: Crystal and structure refinement data for $[NEt_4][F(Cl_2)_3]$ and $[NEt_4][F(Br_2)_3]$.

	$[NEt_4][F(Cl_2)_3]$	$[NEt_4][F(Br_2)_3]$
Empirical formula	C_8Cl_6FN	Br_6C_8FN
Formula weight	341.79	608.55
Temperature/K	100(2)	100(2)
Crystal system	cubic	cubic
Space group	$Pm\bar{3}m$	$Pm\bar{3}m$
a/Å	7.1147(3)	7.5330(3)
Volume/Å^3	360.14(5)	427.47(5)
Z	1	1
ρ_{calc}g/cm^3	1.576	2.364
μ/mm^{-1}	1.175	14.074
F(000)	166.0	274.0
Crystal size/mm^3	0.295 × 0.166 × 0.115	0.381 × 0.341 × 0.305
Radiation	MoK$_\alpha$ (λ = 0.71073)	MoK$_\alpha$ (λ = 0.71073)
2Θ range for data collection/°	5.726 to 56.568	5.408 to 55.06
Index ranges	–9 ≤ h ≤ 9, –9 ≤ k ≤ 9, –9 ≤ l ≤ 9	–9 ≤ h ≤ 9, –9 ≤ k ≤ 9, –9 ≤ l ≤ 9
Reflections collected	5049	36040
Independent reflections	127 [R_{int} = 0.0262, R_{sigma} = 0.0091]	132 [R_{int} = 0.0815, R_{sigma} = 0.0126]
Data/restraints/parameters	127/0/12	132/2/14
Goodness-of-fit on F^2	1.298	1.179
Final R indexes [I>=2σ (I)]	R_1 = 0.0541, wR_2 = 0.1510	R_1 = 0.0241, wR_2 = 0.0561
Final R indexes [all data]	R_1 = 0.0551, wR_2 = 0.1533	R_1 = 0.0241, wR_2 = 0.0561
Largest diff. peak/hole / e Å^{-3}	1.22/–0.98	0.89/–0.46
CCDC	2418343	2418273

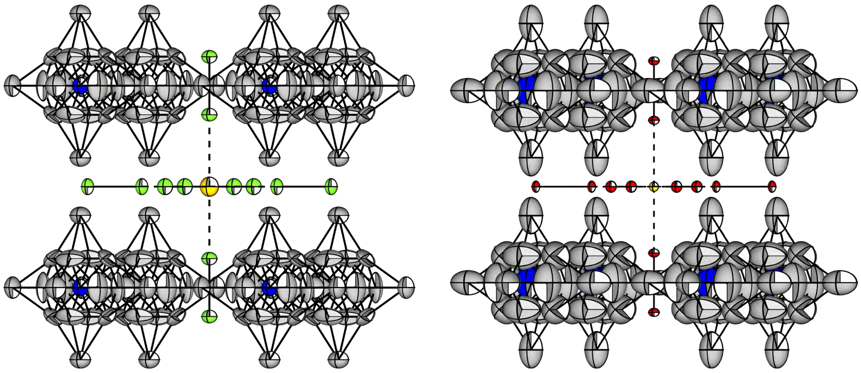

Figure S9: Molecular structures in the solid state of $[NEt_4][F(Cl_2)_3]$ (left) and $[NEt_4][F(Br_2)_3]$ (right). Thermal ellipsoids are set to 50% probability. Nitrogen is blue, fluorine is yellow, chlorine is green, and bromine is red.

The original structure solutions for $[NEt_4][F(Cl_2)_3]$ and $[NEt_4][F(Br_2)_3]$ gave different origins and were refined accordingly. We note a coordinate transformation for all atoms of $[NEt_4][F(Cl_2)_3]$ by –0.5 –0.5 –0.5, as done for the quantum chemical calculations to afford comparability and for an easier visual representation, yields identical refinement parameters after the assembly of fragments.

4. Quantum-chemical calculations:

4.1 Electrostatic potential of halogens, fluorine containing interhalides, $[F(Cl_2)_3]^-$ and $[F(Br_2)_3]^-$:

The electrostatic potential of the halogens, XF, XF_3, XF_5 (X = Cl, Br, I), $[F(Cl_2)_3]^-$ and $[F(Br_2)_3]^-$ plotted onto their electron densities are shown in Figure S10-S13. The molecules were optimized in ORCA 5.0.3 using B3LYP-D3(*BJ*)/def2-QZVPPD, the plots were generated in GAUSSIAN 16 at B3LYP-D3(*BJ*)/def2-QZVPP level of theory and visualized using VMD 1.9.3. Unless stated otherwise, the standard optimization convergence criteria (Opt) as implemented in ORCA 5.0.3 were used, along with the DEFGRID3 and the VeryTightSCF keyword for the calculations.

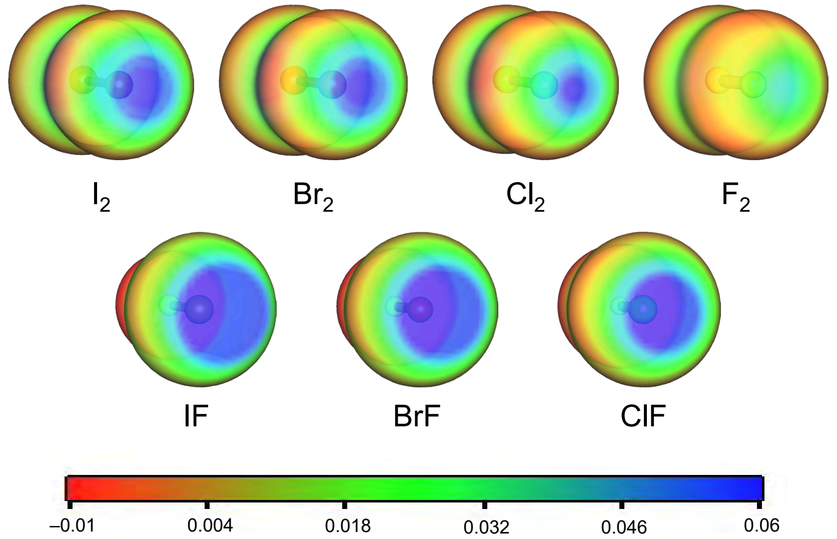

Figure S10: Electrostatic potentials of X_2 and XF with X = F, Cl, Br, I in the range of −0.01 a.u. (red) to 0.06 a.u. (blue) are mapped onto isosurfaces of their electron densities (isosurface value 0.0035 a.u.); calculated at the B3LYP-D3(*BJ*)/def2-QZVPP level of theory (GAUSSIAN 16) in the gas phase.

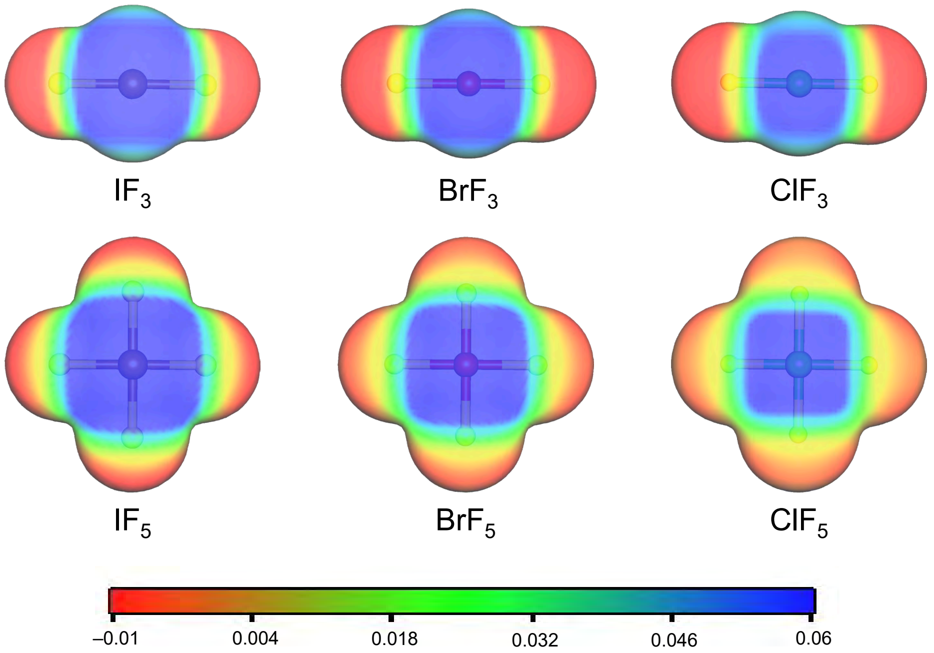

Figure S11: Electrostatic potentials of XF_n with X = Cl, Br, I and n = 3, 5 in the range of −0.01 a.u. (red) to 0.06 a.u. (blue) are mapped onto isosurfaces of their electron densities (isosurface value 0.0035 a.u.); calculated at the B3LYP-D3(*BJ*)/def2-QZVPP level of theory (GAUSSIAN 16) in the gas phase.

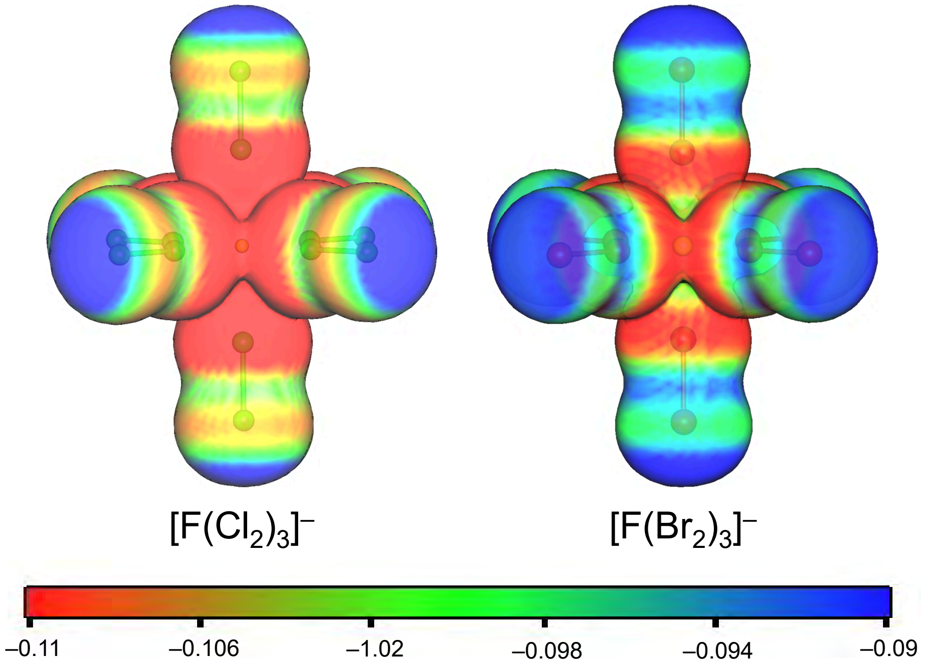

Figure S12: Electrostatic potentials of $[F(X_2)_3]^-$ with X = Cl, Br in the range of −0.11 a.u. (red) to −0.09 a.u. (blue) are mapped onto isosurfaces of their electron densities (isosurface value 0.0035 a.u.); calculated at the B3LYP-D3(*BJ*)/def2-QZVPPD level of theory (GAUSSIAN 16) in the gas phase. The molecules were optimized in ORCA 6.0.1 at B3LYP-D4/def2-QZVPPD level of theory, as stated in chapter 4.2.

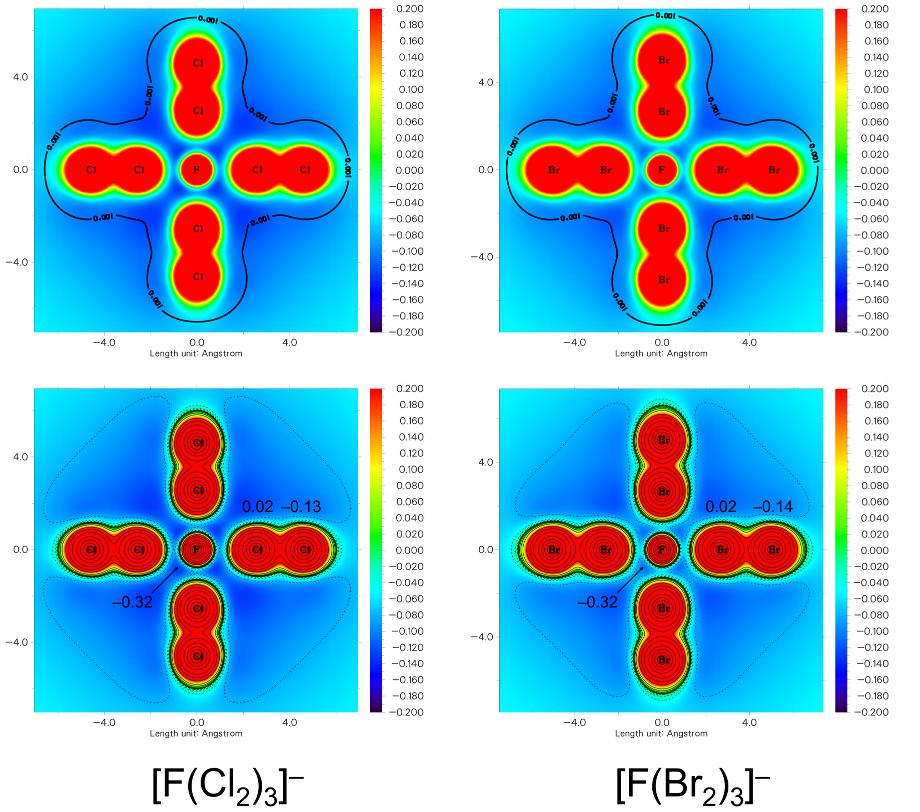

Figure S13: Plot of the electrostatic potential (in a.u.) in the XY plane for $[F(X_2)_3]^-$ with X = Cl, Br in the range of −0.20 a.u. (blue) to 0.20 a.u. (red). The thick black line in the upper plots show the electron density contour line (rho=0.001), whereas small black lines in the lower plots reflect contour lines of the lateral scale and ADCH charges. All plots were determined and graphically represented by MULTIWFN 3.8 with a 200, 200 grid. The RHF wavefunction was calculated based on the optimized structure in the gas phase for the isolated anions at B3LYP-D4/def2-QZVPPD level of theory in ORCA 6.0.1.

4.2 Energy calculations:

All subsequent calculations were performed with ORCA 6.0.1 and the following settings were applied: for all optimisations the rigorous custom convergence criteria (%geom, TolE 1e^{-9}, TolMaxG 1e^{-6}, TolMaxD 1e^{-6}, EnforceStrictConvergence True, end) were used in combinations with DEFGRID3 or refgrid and VeryTightSCF or ExtremSCF cycles. All structures were confirmed as a minimum by means of harmonic frequency calculations. In some cases, the keywords NOCOSX (turns off RIJCOSX) and recalc_hess 10 (recalculating the hessian after 10 geometry optimisation cycels) were used, to ensure convergence and no imaginary frequencies.

Table S3: Gibbs free energy (***ΔG***) in kJ/mol for the addition of one X_2 unit. All calculations were done with B3LYP-D4, PBE0-D4 and M062X with the def2-QZVPPD basis set in ORCA 6.0.1.

Reaction:	Functional	$X_2 = Cl_2$ in kJ/mol	$X_2 = Br_2$ in kJ/mol	$X_2 = I_2$ in kJ/mol
$F^- + X_2 \longrightarrow [F(X_2)]^-$	B3LYP	–130.9	–179.9	–207.9
	PBE0	–134.3	–185.6	–217.8
$[F(X_2)]^- + X_2 \longrightarrow [F(X_2)_2]^-$	B3LYP	–39.8	–33.8	–42.4
	PBE0	–35.3	–32.3	–42.5
$[F(X_2)_2]^- + X_2 \longrightarrow [F(X_2)_3]^-$	B3LYP	–5.5	–9.7	–12.2
	PBE0	–4.1	–8.3	–11.0
$[F(X_2)_3]^- + X_2 \longrightarrow [F(X_2)_4]^-$	B3LYP	14.9	15.2	12.6
	PBE0	15.5	16.2	15.8
$[F(X_2)_4]^- + X_2 \longrightarrow [F(X_2)_5]^-$	B3LYP	21.0	22.0	30.8 [a)]
	PBE0	21.4	25.1	
$[F(X_2)_5]^- + X_2 \longrightarrow [F(X_2)_6]^-$	B3LYP	27.6	31.0	30.1 [a)]
	PBE0	31.0	33.9	

[a)] M062X/def2-QZVPPD was used without D4, since no minimum could be found for $[F(I_2)_5]^-$ with PBE0-D4 and B3LYP-D4 with the def2-QZVPPD basis set.

Table S4: Gibbs free energy (***ΔG***) in kJ/mol for the addition of one X_2 unit. All calculations were done with B3LYP-D4 and PBE0-D4 with the def2-QZVPPD basis set in ORCA 6.0.1.

Reaction:	Functional	X = Cl in kJ/mol	X = Br in kJ/mol	X = I in kJ/mol
$X^- + X_2 \longrightarrow [X(X_2)]^-$	B3LYP	–94.9	–110.0	–112.3
	PBE0	–91.6	–110.2	–115.9
$[X(X_2)]^- + X_2 \longrightarrow [X(X_2)_2]^-$	B3LYP	–17.3	–30.6	–37.6
	PBE0	–15.6	–30.3	–39.8
$[X(X_2)_2]^- + X_2 \longrightarrow [X(X_2)_3]^-$	B3LYP	1.2	–4.4	–8.2
	PBE0	1.4	–3.8	–9.4
$[X(X_2)_3]^- + X_2 \longrightarrow [X(X_2)_4]^-$	B3LYP	14.3	12.6	10.9
	PBE0	13.9	12.4	11.0
$[X(X_2)_4]^- + X_2 \longrightarrow [X(X_2)_5]^-$	B3LYP	18.5	16.9	13.6
	PBE0	17.9	19.3	12.8
$[X(X_2)_5]^- + X_2 \longrightarrow [X(X_2)_6]^-$	B3LYP	25.4	26.0	17.3
	PBE0	28.6	25.0	23.8

4.3 Quantum-chemically optimized structures:

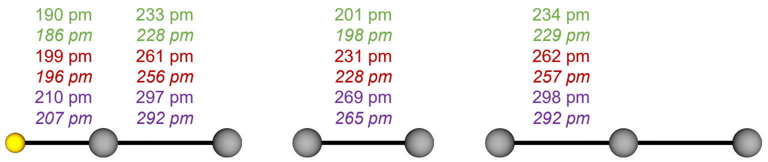

Figure S14: Optimized structures for the isolated $[F(X_2)]^-$ anion, X_2 molecule and $[X(X_2)]^-$ anion calculated at the B3LYP-D4/def2-QZVPPD and PBE0-D4/def2-QZVPPD (italics) level of theory. The fluorine atom is shown in yellow and the grey atoms are representative of chlorine atoms (bond lengths in green), bromine atoms (bond lengths in red) and iodine atoms (bond lengths in purple).

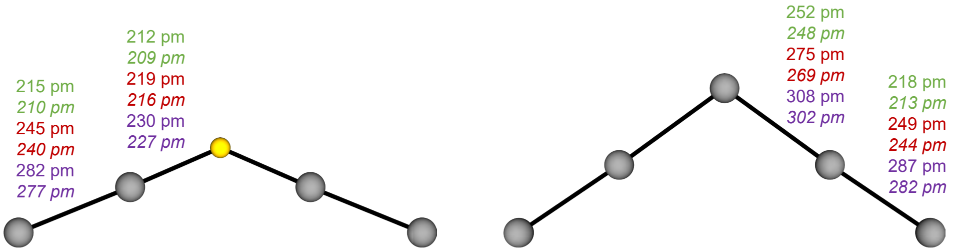

Figure S15: Optimized structures for the isolated $[F(X_2)_2]^-$ and $[X(X_2)_2]^-$ anion calculated at the B3LYP-D4/def2-QZVPPD and PBE0-D4/def2-QZVPPD (italics) level of theory. The fluorine atom is shown in yellow and the grey atoms are representative of chlorine atoms (bond lengths in green), bromine atoms (bond lengths in red) and iodine atoms (bond lengths in purple). The shown anions have a planar C_{2v} structure.

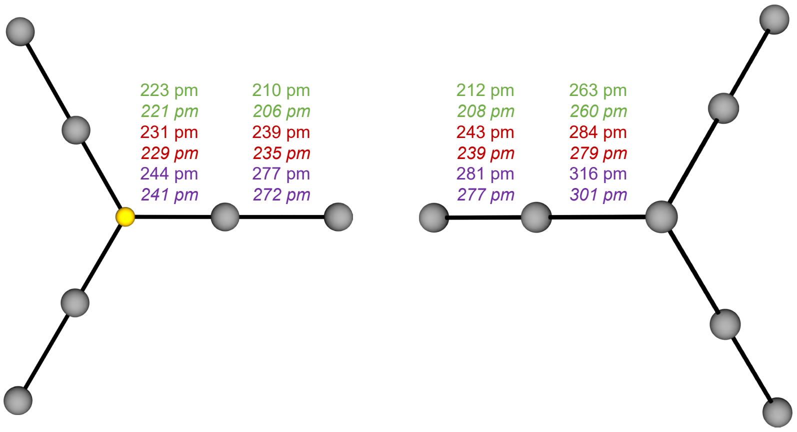

Figure S16: Optimized structures for the isolated $[F(X_2)_3]^-$ and $[X(X_2)_3]^-$ anion calculated at the B3LYP-D4/def2-QZVPPD and PBE0-D4/def2-QZVPPD (italics) level of theory. The fluorine atom is shown in yellow and the grey atoms are representative of chlorine atoms (bond lengths in green), bromine atoms (bond lengths in red) and iodine atoms (bond lengths in purple). The shown anions have a planar D_{3h} structure.

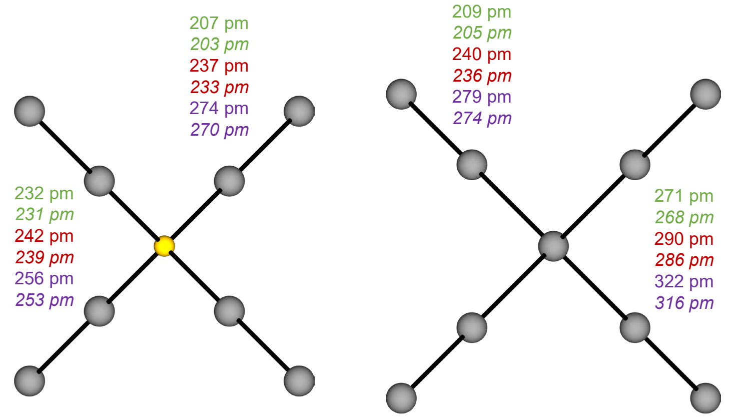

Figure S17: Optimized structures for the isolated $[F(X_2)_4]^-$ and $[X(X_2)_4]^-$ anion calculated at the B3LYP-D4/def2-QZVPPD and PBE0-D4/def2-QZVPPD (italics) level of theory. The fluorine atom is shown in yellow and the grey atoms are representative of chlorine atoms (bond lengths in green), bromine atoms (bond lengths in red) and iodine atoms (bond lengths in purple). The shown anions have a T_d structure.

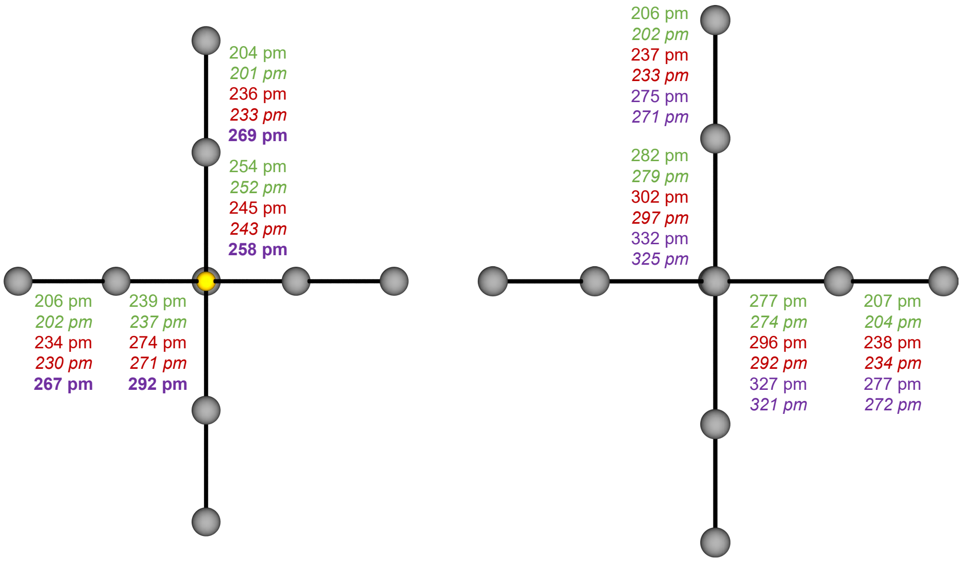

Figure S18: Optimized structures for the isolated $[F(X_2)_5]^-$ and $[X(X_2)_5]^-$ anion calculated at the B3LYP-D4/def2-QZVPPD, PBE0-D4/def2-QZVPPD (italics) and M062X/def2-QZVPPD (bold) level of theory. The fluorine atom is shown in yellow and the grey atoms are representative of chlorine atoms (bond lengths in green), bromine atoms (bond lengths in red) and iodine atoms (bond lengths in purple). The shown anions have a D_{3h} structure.

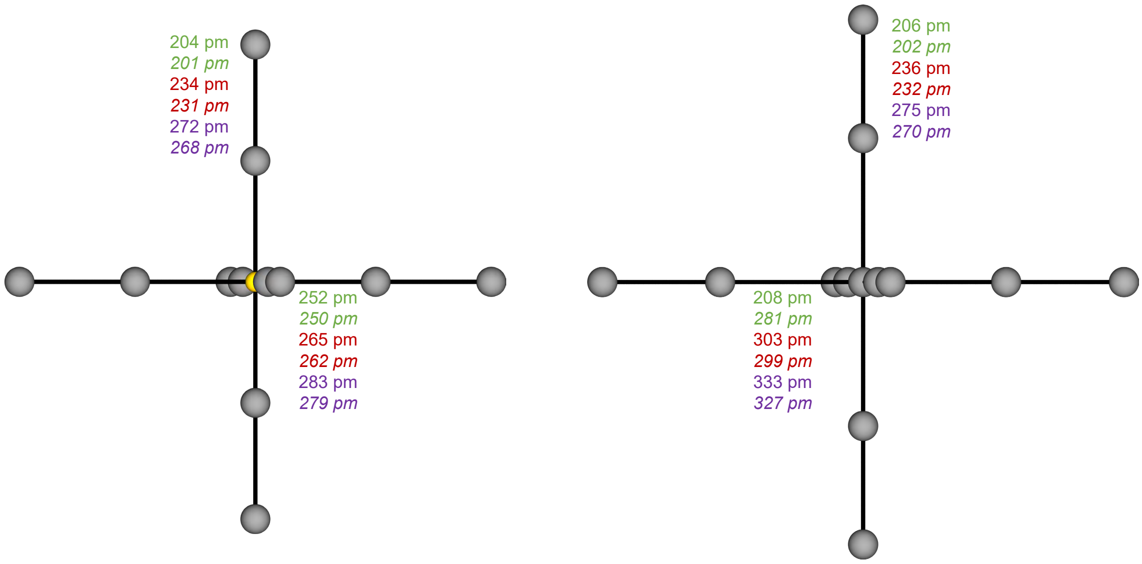

Figure S19: Optimized structures for the isolated $[F(X_2)_6]^-$ and $[X(X_2)_6]^-$ anion calculated at the B3LYP-D4/def2-QZVPPD and PBE0-D4/def2-QZVPPD (italics) level of theory. The fluorine atom is shown in yellow and the grey atoms are representative of chlorine atoms (bond lengths in green), bromine atoms (bond lengths in red) and iodine atoms (bond lengths in purple). The shown anions have a O_h structure.

4.4 XYZ files optimized with B3LYP-D4/def2-QZVPPD (Orca 6.0.1):

Cl^{-}

Final single point energy: –460.248528080142 Eh

Total enthalpy: –460.2461676 Eh

Gibbs free energy: –460.26357022 Eh

Cl_2

Zero point energy: 0.00122624 Eh

Final single point energy: –920.323704556492 Eh

Total enthalpy: –920.31897633 Eh

Final Gibbs free energy: –920.34497733 Eh

Cl	-7.19888637285581	2.52812000000000	0.00000000000000
Cl	-5.18897362714418	2.52812000000000	0.00000000000000

$[Cl_3]^{-}$

Zero point energy: 0.00181918 Eh

Final single point energy: –1380.620013884763 Eh

Total enthalpy: –1380.61263039 Eh

Final Gibbs free energy: –1380.64468057 Eh

Cl	-5.64208608129433	1.65386091435653	0.00000000727162
Cl	-3.30037333878010	1.60279030553428	-0.00000001994296
Cl	-0.95866057992555	1.55171878010918	0.00000001267135

$[Cl(Cl_2)_2]^{-}$

Zero point energy: 0.00364200 Eh

Final single point energy: –2300.964596100365 Eh

Total enthalpy: –2300.95167809 Eh

Final Gibbs free energy: –2300.99625213 Eh

Cl	-0.00002991570237	1.76385839853286	-0.00000014314488
Cl	-0.00000179499264	0.30927907736036	2.05872660320097
Cl	0.00002168300020	-0.90106830631489	3.87167907396065
Cl	-0.00000150928010	0.30927911035675	-2.05872653181885
Cl	0.00002153697492	-0.90106827993508	-3.87167900219787

$[Cl(Cl_2)_3]^–$

Zero point energy: 0.00539670 Eh

Final single point energy: –3221.303138683962 Eh

Total enthalpy: –3221.28416577 Eh

Final Gibbs free energy: –3221.34077280 Eh

Cl	-1.75843338689661	1.64300984196825	0.20337887405943
Cl	-3.17743909553930	2.97913303946474	-0.62876868829710
Cl	-0.53925388964747	-2.35054441944693	0.20480998053520
Cl	-0.97049023814279	-4.25120358288790	-0.62770743963831
Cl	2.29458391670868	0.70439556268335	0.20296724211121
Cl	4.15590744881641	1.27958825853509	-0.63095227098494
Cl	-0.00487475529891	-0.00437870031659	1.27627230421451

$[Cl(Cl_2)_4]^–$

Zero point energy: 0.00711952 Eh

Final single point energy: –4141.638747010251 Eh

Total enthalpy: –4141.61367123 Eh

Final Gibbs free energy: –4141.68029856 Eh

Cl	-1.56346969172274	-1.56345971005432	1.56351023985541
Cl	-2.77020673942055	-2.77018548007262	2.77027948198391
Cl	1.56346826341026	1.56346194041184	1.56350930173207
Cl	2.77019949390413	2.77019143119082	2.77028060869335
Cl	1.56347102379567	-1.56346241470747	-1.56351203546509
Cl	2.77020238882189	-2.77018900389966	-2.77028589438970
Cl	-1.56346677207413	1.56345930143788	-1.56350663567522
Cl	-2.77020147281369	2.77018540868706	-2.77027697768881
Cl	0.00000350609917	-0.00000147299350	0.00000191095408

$[Cl(Cl_2)_5]^–$

Zero point energy: 0.00871756 Eh

Final single point energy: –5061.971560869727 Eh

Total enthalpy: –5061.9403663 Eh

Final Gibbs free energy: –5062.01822842 Eh

Cl	0.01779878526565	-0.00000391721666	0.00000358940454
Cl	0.02304452979663	-0.00000439052271	2.82064265838328
Cl	0.02638259545111	-0.00000576568392	4.88102462196530

Cl	0.02304770735401	0.00000021982472	-2.82063310184995
Cl	0.02638572933887	0.00000129053682	-4.88101522836985
Cl	-1.40453535226766	2.37239625365184	-0.00000300013396
Cl	-2.47600244211386	4.14765290646740	-0.00001004585105
Cl	2.78565555196545	0.00000324614011	-0.00000242848607
Cl	4.85877458193597	0.00000829037938	-0.00000841608178
Cl	-1.40453947472141	-2.37239747758692	0.00000068222791
Cl	-2.47601221400478	-4.14765065599007	0.00000066879170

$[Cl(Cl_2)_6]^-$

Zero point energy: 0.01030373 Eh

Final single point energy: –5982.304673456163 Eh

Total enthalpy: –5982.26734555 Eh

Final Gibbs free energy: –5982.35354860 Eh

Cl	-0.00000425763852	-0.00000241310344	-2.83954537414844
Cl	-0.00000504221526	-0.00000571643848	-4.89493038215937
Cl	0.00000569818101	2.83955930464123	0.00000773233826
Cl	0.00001203830075	4.89494398389144	0.00000917699169
Cl	-0.00000776428291	-0.00000403210912	2.83955065235428
Cl	-0.00001214681026	-0.00000998848825	4.89493604104791
Cl	0.00000714444764	-2.83955193645604	0.00000921753765
Cl	0.00001462750144	-4.89493625054064	0.00001352663895
Cl	2.83955283398421	0.00000101280977	-0.00001013986547
Cl	4.89493726867371	-0.00000147570206	-0.00002239441398
Cl	-2.83955668360442	0.00000206075622	-0.00000687399407
Cl	-4.89494141342969	0.00000134460574	-0.00001515017765
Cl	-0.00000230310769	0.00000410613363	0.00000396785025

F^-

Final single point energy: –99.868155321036 Eh

Total enthalpy: –99.86579484 Eh

Gibbs free energy: –99.88231389Eh

$[F(Cl_2)]^-$

Zero point energy: 0.00200641 Eh

Final single point energy: –1020.261400793335 Eh

Total enthalpy: –1020.254303 Eh

Final Gibbs free energy: –1020.27713064 Eh

F	-5.33879424287690	1.64723200738874	0.00000000372765
Cl	-3.44446088880803	1.60594585182644	-0.00000000764691
Cl	-1.11786486831504	1.55519214078483	0.00000000391925

$[F(Cl_2)_2]^-$

Zero point energy: 0.00447487 Eh

Final single point energy: –1940.607973097193 Eh

Total enthalpy: –1940.59475239 Eh

Final Gibbs free energy: –1940.63725067 Eh

F	-0.00002084691039	1.33626188429214	0.00000013226931
Cl	-0.00000167293292	0.30854337401157	1.84833590164910
Cl	0.00001705708352	-0.68653436409855	3.75070961387668
Cl	-0.00000151832493	0.30854355443304	-1.84833607526577
Cl	0.00001698108475	-0.68653444863819	-3.75070957252934

$[F(Cl_2)_3]^-$

Zero point energy: 0.00634689 Eh

Final single point energy: –2860.948894748443 Eh

Total enthalpy: –2860.92956997 Eh

Final Gibbs free energy: –2860.98430450 Eh

Cl	1.83128623635191	1.27748810979580	-0.00019554134700
Cl	3.54988990340499	2.47508841557670	0.00054194825613
Cl	0.19061473973585	-2.22324932861997	-0.00020093662134
Cl	0.36931568431366	-4.31027836070313	0.00054285102539
Cl	-2.02183956699225	0.94650147888543	-0.00020709587035
Cl	-3.91943256666850	1.83351959001607	0.00054744038765
F	0.00016556985434	0.00093009404911	-0.00103964983048

$[F(Cl_2)_4]^-$

Zero point energy: 0.00832054 Eh

Final single point energy: –3781.286864989871 Eh

Total enthalpy: –3781.26137201 Eh

Final Gibbs free energy: –3781.32360386 Eh

Cl	-1.34190650756385	-1.34190701221991	-1.34191042534111

Cl	-2.53648180858064	-2.53648326366132	-2.53648977593962
Cl	1.34190738572267	1.34190719574363	-1.34191149122049
Cl	2.53648318127767	2.53648211718992	-2.53649147094633
Cl	-1.34190648662516	1.34190709386297	1.34191159184863
Cl	-2.53648323350604	2.53648132603038	2.53649157116255
Cl	1.34190615668499	-1.34190589284161	1.34191038154632
Cl	2.53648067326772	-2.53648295641241	2.53648989825269
F	0.00000064032264	0.00000139330836	-0.00000027836264

$[F(Cl_2)_5]^-$

Zero point energy: 0.00988399 Eh

Final single point energy: –4701.617786284827 Eh

Total enthalpy: –4701.58619118 Eh

Final Gibbs free energy: –4701.66059828 Eh

F	0.01120640139475	0.00000416198005	-0.00000094944832
Cl	0.01600276591893	0.00001050106712	-2.54333692911643
Cl	0.01952907842149	0.00001534664067	-4.58359339117343
Cl	0.01600075974453	0.00000932621534	2.54333705338562
Cl	0.01952137879032	0.00001383198830	4.58359328236958
Cl	-1.20792727601201	-2.05311025245090	0.00000045701419
Cl	-2.26289250230315	-3.82149678077049	0.00000475537233
Cl	2.40021264933013	-0.00001411453083	-0.00000198946421
Cl	4.45921249029112	-0.00003039056062	-0.00000438336457
Cl	-1.20794411528521	2.05310959974980	-0.00000051527292
Cl	-2.26292163129091	3.82148877067154	0.00000260969817

$[F(Cl_2)_6]^-$

Zero point energy: 0.01147624 Eh

Final single point energy: –5621.951140251445 Eh

Total enthalpy: –5621.91341894 Eh

Final Gibbs free energy: –5621.99507803 Eh

Cl	0.00000065887998	0.00000012512971	-2.52132523922169
Cl	0.00000010106179	0.00000151719609	-4.56252969776161
Cl	-0.00000061793235	2.52132807019004	-0.00000271562500
Cl	-0.00000264117980	4.56253261594823	-0.00000406613636
Cl	0.00000123860668	0.00000062403519	2.52132397204517

Cl	0.00000178459978	0.00000221758295	4.56252859123575
Cl	0.00000075244908	-2.52133135362514	-0.00000265796305
Cl	0.00000015129520	-4.56253574661211	-0.00000507423719
Cl	2.52132757258439	-0.00000073655199	0.00000320009581
Cl	4.56253228776297	0.00000020062785	0.00000620620522
Cl	-2.52132904128871	0.00000103649044	0.00000271461583
Cl	-4.56253342056892	0.00000277060145	0.00000447068199
F	0.00000117372992	-0.00000134101271	0.00000029606513

Br^-

Final single point energy: –2574.182532918356 Eh

Total enthalpy: –2574.18018135 Eh

Final Gibbs free energy: –2574.19873479 Eh

Br_2

Zero point energy: 0.00072038

Final single point energy: –5148.191997879992 Eh

Total enthalpy: –5148.18757254 Eh

Final Gibbs free energy: –5148.21611961 Eh

Br	-7.35041730080042	2.52812000000000	0.00000000000000
Br	-5.03744269919957	2.52812000000000	0.00000000000000

$[Br_3]^-$

Zero point energy: 0.00113690 Eh

Final single point energy: –7722.428460066876 Eh

Total enthalpy: –7722.42125314 Eh

Final Gibbs free energy: –7722.45675397 Eh

Br	-5.91567041469165	1.65990300733534	0.00000002409479
Br	-3.30037352904366	1.60278979714785	-0.00000003647183
Br	-0.68507605626467	1.54567719551681	0.00000001237701

$[Br(Br_2)_2]^-$

Zero point energy: 0.00225684 Eh

Final single point energy: –12870.647281894899 Eh

Total enthalpy: –12870.63475463 Eh

Final Gibbs free energy: –12870.68452805 Eh

Br	-0.00003244766228	1.90376824988435	0.00000057928090
Br	-0.00000191897822	0.34018869129340	2.25579106830218
Br	0.00002299012249	-1.00193281009489	4.35286647630378
Br	-0.00000131190139	0.34018910578231	-2.25579151525935
Br	0.00002268841940	-1.00193323686517	-4.35286660862751

$[Br(Br_2)_3]^-$

Zero point energy: 0.00335881 Eh

Final single point energy: –18018.857581258100 Eh

Total enthalpy: –18018.83924105 Eh

Final Gibbs free energy: –18018.90231231 Eh

Br	-1.38560632733622	-2.16377151718828	-0.24095972102292
Br	-2.58614765571634	-4.04827184619838	0.71809499816886
Br	2.58672159841794	-0.13145828255451	-0.24316845203796
Br	4.81902873503114	-0.26757058621660	0.71113866132718
Br	-1.19762149615692	2.30783162443067	-0.24249074187902
Br	-2.24102017791827	4.28720422178447	0.70738077037833
Br	0.00464532467866	0.01603638594264	-1.40999551593447

$[Br(Br_2)_4]^-$

Zero point energy: 0.00442094 Eh

Final single point energy: –23167.063539122886 Eh

Total enthalpy: –23167.03936596 Eh

Final Gibbs free energy: –23167.11362688 Eh

Br	-1.67445919009063	-1.67445689529662	-1.67443107025457
Br	-3.06069326041235	-3.06066292718575	-3.06061353769309
Br	1.67446148627264	-1.67446313081692	1.67443966575165
Br	3.06066768347149	-3.06067518626259	3.06064325029959
Br	1.67445902211897	1.67446279061199	-1.67443531707011
Br	3.06066168038585	3.06069714681606	-3.06061953402926
Br	-1.67444622464248	1.67444712184350	1.67441891046425
Br	-3.06066614749388	3.06066319304340	3.06060472948294
Br	0.00001495039038	-0.00001211275309	-0.00000709695139

$[Br(Br_2)_5]^-$

Zero point energy: 0.00528031 Eh

Final single point energy: –28315.265752804156 Eh

Total enthalpy: –28315.23576674 Eh

Final Gibbs free energy: –28315.32332373 Eh

Br	-1.48425819352690	-2.55224353810045	-0.00001266850411
Br	-2.68964310506107	-4.60812197922197	-0.00002633620576
Br	-1.48422605925078	2.55224983727605	0.00000522278349
Br	-2.68958989426474	4.60814171847064	0.00001048694417
Br	2.96478913757512	0.00002720740321	0.00000960033091
Br	5.34793842049211	0.00005758686144	0.00002434201983
Br	0.00719646178853	-0.00002905106418	-3.01676422627264
Br	0.00602240684586	-0.00004773403355	-5.38440516064499
Br	0.00721301335618	-0.00001260459588	3.01675981649898
Br	0.00605576823286	-0.00001747425262	5.38440063391958
Br	0.00850204281281	-0.00000396874272	-0.00000171086947

$[Br(Br_2)_6]^-$

Zero point energy: 0.00615076 Eh

Final single point energy: –33463.468487259859 Eh

Total enthalpy: –33463.43266778 Eh

Final Gibbs free energy: –33463.52954245 Eh

Br	-0.00001030271384	-0.00000361475740	3.03267055279193
Br	-0.00002735357588	-0.00000851376734	5.39516545061191
Br	-3.03265243302956	0.00000167312362	0.00002193920849
Br	-5.39514643878916	0.00000193456124	0.00003092465164
Br	-0.00000318255911	-0.00000107797203	-3.03265079242893
Br	-0.00001103841742	-0.00000135146607	-5.39514480355356
Br	3.03266901518869	0.00000348412879	0.00001456107490
Br	5.39516385027514	0.00000772275560	0.00001475750509
Br	0.00000223265159	-3.03266078683074	-0.00001881240117
Br	-0.00000284624613	-5.39515523411972	-0.00004369444172
Br	0.00000584976427	3.03266070056841	-0.00002193165708
Br	0.00000192412836	5.39515511468065	-0.00005052859660
Br	0.00001072332305	-0.00000005090501	0.00001237723512

$[F(Br_2)]^-$

Zero point energy: 0.00197938 Eh

Final single point energy: –5248.141540750190 Eh

Total enthalpy: –5248.13405182 Eh

Final Gibbs free energy: –5248.16694811 Eh

F	-2.93989136547547	0.00002885938518	-0.00000000230520
Br	-0.94855718397547	0.00000758466584	0.00000000461045
Br	1.65629042939995	-0.00000496754802	-0.00000000230525

$[F(Br_2)_2]^-$

Zero point energy: 0.00324213 Eh

Final single point energy: –10396.361800975896 Eh

Total enthalpy: –10396.34899824 Eh

Final Gibbs free energy: –10396.39592611 Eh

F	0.00000001210938	0.00000010219021	-1.43654974521296
Br	-0.00000006649259	1.95254454246042	-0.44057892766925
Br	0.00000003141013	4.15877633007154	0.61352526183519
Br	0.00000004294153	-1.95254457697775	-0.44057864465217
Br	-0.00000001996845	-4.15877639774442	0.61352517057119

$[F(Br_2)_3]^-$

Zero point energy: 0.00441307 Eh

Final single point energy: –15544.573910951773 Eh

Total enthalpy: –15544.55527934 Eh

Final Gibbs free energy: –15544.61575238 Eh

Br	-4.69923403329391	-0.23901142045107	0.00001335561670
Br	-2.30856449728165	-0.11556936453580	-0.00000635312119
F	0.00195512618726	0.00280805069897	-0.00002453621034
Br	1.05699185475361	2.06117773956078	-0.00000612946431
Br	1.25325111925222	-1.94297543931644	-0.00000567974034
Br	2.14943504933833	4.19148595845520	0.00001334264715
Br	2.54616538104513	-3.95791552441163	0.00001303784433

$[F(Br_2)_4]^-$

Zero point energy: 0.00565440 Eh

Final single point energy: –20692.781802590678 Eh

Total enthalpy: –20692.75731012 Eh

Final Gibbs free energy: –20692.82609476 Eh

Br	-1.39414749393324	1.39414687322652	-1.39414652569558
Br	-2.76147905907015	2.76147503975266	-2.76147445919932
Br	-1.39414634860252	-1.39414616396853	1.39414559678540
Br	-2.76147688580427	-2.76147528009101	2.76147355688835
Br	1.39414732501206	-1.39414666080670	-1.39414601882707
Br	2.76147671905695	-2.76147700207455	-2.76147393641785
Br	1.39414762440844	1.39414712175907	1.39414626043072
Br	2.76147748807230	2.76147505576709	2.76147611482587
F	0.00000063344243	0.00000101901745	-0.00000058620851

$[F(Br_2)_5]^-$

Zero point energy: 0.00630740 Eh

Final single point energy: –25840.980652079805 Eh

Total enthalpy: –25840.95039642 Eh

Final Gibbs free energy: –25841.03384384 Eh

Br	1.22864890735252	-2.11874421875191	0.00000163740526
Br	2.41779589755497	-4.16175069973150	0.00000039481705
Br	1.22864887966571	2.11874472891588	0.00000030251232
Br	2.41779608313608	4.16175109078453	-0.00000192751430
Br	-2.45338870997197	-0.00000100678297	-0.00000041428970
Br	-4.81728301717181	-0.00000254688561	-0.00000352809129
Br	-0.00426623040470	0.00000125591975	2.74291666129982
Br	-0.00549213560435	0.00000179310488	5.07896187566157
Br	-0.00426414684039	-0.00000063839804	-2.74291582856452
Br	-0.00548780675702	-0.00000203999468	-5.07896081211223
F	-0.00270772095904	0.00000028181965	0.00000263887604

$[F(Br_2)_6]^-$

Zero point energy: 0.00722984 Eh

Final single point energy: –30989.183042783730 Eh

Total enthalpy: –30989.14697941 Eh

Final Gibbs free energy: –30989.23815842 Eh

Br	0.00000000156657	2.64546202568155	-0.00000018424847
Br	0.00000001811838	4.98691001458547	-0.00000012481320
Br	-0.00000009448597	-0.00000028109384	2.64546188373788
Br	-0.00000008048335	-0.00000032863473	4.98690990719309

Br	-0.00000022199716	-2.64546217561942	0.00000008101482
Br	-0.00000038371641	-4.98691024609813	0.00000119246181
Br	0.00000011007992	0.00000008856129	-2.64546220297997
Br	0.00000045491042	0.00000093326262	-4.98691023812548
Br	2.64546270086406	-0.00000017723228	-0.00000019146827
Br	4.98691067278197	0.00000061758905	-0.00000015565189
Br	-2.64546254543634	-0.00000002321305	-0.00000001475094
Br	-4.98691052507602	0.00000001545343	0.00000039451516
F	-0.00000010712606	-0.00000046324195	-0.00000034688452

I^-

Final single point energy: –297.8034574468 Eh

Total enthalpy: –297.80108069 Eh

Gibbs free energy: –297.82028933 Eh

I_2

Zero point energy: 0.00048553 Eh

Final single point energy: –595.443862022743 Eh

Total enthalpy: –595.43953136 Eh

Final Gibbs free energy: –595.46978210 Eh

I	-1.34495486009350	0.00000000000000	0.00000000000000
I	1.34495486009350	0.00000000000000	0.00000000000000

$[I_3]^-$

Zero point energy: 0.00078784 Eh

Final single point energy: –893.302428727536 Eh

Total enthalpy: –893.29528444 Eh

Final Gibbs free energy: –893.33285582 Eh

I	-2.98094587095567	0.00000019010385	-0.00000000051143
I	-0.00000000155365	-0.00000038020770	0.00000000102286
I	2.98094587250931	0.00000019010385	-0.00000000051143

$[I(I_2)_2]^-$

Zero point energy: 0.00159056 Eh

Final single point energy: –1488.776213725257 Eh

Total enthalpy: –1488.76380803 Eh

Final Gibbs free energy: –1488.81695269 Eh

I	0.00000011070275	0.00000005788336	-2.09320884721525
I	2.49409021626122	-0.00000006685571	-0.27916331882313
I	4.87164902156579	0.00000003537554	1.32576780574950
I	-2.49409047138334	-0.00000005645295	-0.27916356528213
I	-4.87164887714641	0.00000003004975	1.32576792557101

$[I(I_2)_3]^-$

Zero point energy: 0.00237675 Eh

Final single point energy: –2084.240907978948 Eh

Total enthalpy: –2084.22276456 Eh

Final Gibbs free energy: –2084.28985183 Eh

I	-0.99780284286150	-2.65523393158394	0.32030708814775
I	-1.89399917589932	-5.02943684034656	-0.89521816989057
I	2.79154551253286	0.46899935346990	0.31851428729986
I	5.29269712711928	0.89884061738324	-0.89668753413883
I	-1.79339671985245	2.18098857321880	0.31819098346276
I	-3.40102552420430	4.14287829919539	-0.89949125045303
I	0.00198162316543	-0.00703607133683	1.73438459557405

$[I(I_2)_4]^-$

Zero point energy: 0.00306734 Eh

Final single point energy: –2679.700277416188 Eh

Total enthalpy: –2679.67639559 Eh

Final Gibbs free energy: –2679.75548758 Eh

I	1.85741309347919	-1.85741190467842	-1.85741333202544
I	3.46552362421864	-3.46552499435818	-3.46552851761261
I	-1.85741126548711	-1.85740941230258	1.85741044919897
I	-3.46552837010619	-3.46551957878988	3.46552216245760
I	1.85741277497740	1.85741171176902	1.85741303314776
I	3.46552321611611	3.46552641558402	3.46552695013548
I	-1.85741060418331	1.85740886734451	-1.85740963266057
I	-3.46552660492645	3.46551964789227	-3.46552162933618
I	0.00000413591272	-0.00000075245976	0.00000051669601

$[I(I_2)_5]^-$

Zero point energy: 0.00370618 Eh

Final single point energy: –3275.156505978600 Eh

Total enthalpy: –3275.12688475 Eh

Final Gibbs free energy: –3275.22008090 Eh

I	1.64590327773976	-2.81800259304550	0.00000096005157
I	3.05046269695316	-5.20104161221986	0.00000339856666
I	-3.28282459781434	-0.00000185351104	0.00000021761280
I	-6.04861596722287	-0.00000255607553	0.00000066200470
I	1.64590407484542	2.81799980379115	-0.00000045073856
I	3.05046366028214	5.20103851652544	-0.00000053623138
I	-0.01239255988814	0.00000258953198	-3.32025074328640
I	-0.01212304542402	0.00000567284147	-6.07083807147491
I	-0.01239207689372	0.00000095436820	3.32024916365258
I	-0.01212239734091	0.00000253030565	6.07083642133372
I	-0.01226306523649	-0.00000145251197	-0.00000102149077

$[I(I_2)_6]^-$

Zero point energy: 0.00428304 Eh

Final single point energy: –3870.613179634716 Eh

Total enthalpy: –3870.57781707 Eh

Final Gibbs free energy: –3870.68328934 Eh

I	-3.33383860724797	-0.00000006256577	0.00000004938731
I	-6.07837853288080	-0.00000025934097	0.00000017082267
I	-0.00000007551649	0.00000005613301	-3.33383862615521
I	-0.00000012729863	-0.00000000090815	-6.07837844605475
I	3.33383851588783	0.00000003452060	-0.00000000958329
I	6.07837833329005	-0.00000006459166	-0.00000000493113
I	-0.00000016031362	0.00000006146190	3.33383825730251
I	-0.00000028888722	-0.00000005554260	6.07837810658475
I	0.00000010516904	-3.33383836934043	0.00000001680622
I	0.00000023485063	-6.07837818956492	0.00000026279072
I	0.00000019690831	3.33383842279798	0.00000006704499
I	0.00000043253269	6.07837827808596	0.00000018352432
I	-0.00000002649383	0.00000014885506	-0.00000002753910

$[F(I_2)]^-$

Zero point energy: 0.00173545 Eh

Final single point energy: –695.404150037001 Eh

Total enthalpy: –695.39671417 Eh

Final Gibbs free energy: –695.43128122 Eh

F	3.33795883812243	-0.00004198848141	0.00000002862867
I	1.23706932779403	-0.00002007611661	-0.00000005725733
I	-1.73678408314046	0.00001134862402	0.00000002862866

$[F(I_2)_2]^-$

Zero point energy: 0.00265407 Eh

Final single point energy: –1290.880395341000 Eh

Total enthalpy: –1290.86773931 Eh

Final Gibbs free energy: –1290.91722804 Eh

F	0.00000003334376	-0.00000003654465	1.39377364884631
I	2.11087393936547	0.00000004303514	0.47746859548914
I	4.72239658052083	-0.00000001980915	-0.58181840784608
I	-2.11087376482410	0.00000002512134	0.47746877804605
I	-4.72239678840596	-0.00000001180269	-0.58181854254341

$[F(I_2)_3]^-$

Zero point energy: 0.00349721 Eh

Final single point energy: –1886.346491791418 Eh

Total enthalpy: –1886.32810108 Eh

Final Gibbs free energy: –1886.39164491 Eh

I	2.42239937579832	-0.29655598307824	-0.00000004301665
I	5.16864732739953	-0.64330036169899	0.00000414326986
I	-0.95559083207313	2.24457677031051	-0.00000008051455
I	-2.04623352093588	4.78859772150148	0.00000416376693
I	-1.46302743950860	-1.94381873761771	-0.00000018001391
I	-3.12989309667563	-4.15343330209775	0.00000420629966
F	0.00369818599439	0.00393389268070	-0.00000400710834

$[F(I_2)_4]^-$

Zero point energy: 0.00427019 Eh

Final single point energy: –2481.807524212097 Eh

Total enthalpy: –2481.7834014 Eh

Final Gibbs free energy: –2481.85663275 Eh

I	1.47495284256981	-1.47495365108832	1.47495284242455
I	3.05815342548710	-3.05815503436666	3.05815351340137
I	-1.47495338398187	-1.47495366508549	-1.47495305835442
I	-3.05815445363611	-3.05815585207121	-3.05815257781776
I	-1.47495372756234	1.47495362320637	1.47495352564219
I	-3.05815487729713	3.05815408927474	3.05815459771240
I	1.47495432509048	1.47495454213590	-1.47495395481019
I	3.05815580227273	3.05815532405675	-3.05815441280653
F	0.00000004515031	0.00000062203092	-0.00000047729762

$[F(I_2)_6]^-$

Zero point energy: 0.00501796 Eh

Final single point energy: –3672.713035218814 Eh

Total enthalpy: –3672.67753923 Eh

Final Gibbs free energy: –3672.77733711 Eh

I	2.82874495715284	-0.00000074617233	0.00000023814617
I	5.54580429077552	-0.00000063558676	-0.00000067942157
I	0.00000328072851	2.82874498305864	0.00000103329405
I	-0.00000691583194	5.54580387286087	0.00000038910020
I	-2.82874297898235	0.00000138747098	0.00000016394763
I	-5.54580164116996	-0.00000148383870	-0.00000018953650
I	0.00000101669605	-2.82874403207449	-0.00000068620178
I	-0.00000536328323	-5.54580282568203	-0.00000059783071
I	0.00000263840258	-0.00000028996594	2.82874104989600
I	-0.00000494756969	-0.00000055673619	5.54580026970377
I	0.00000281327732	0.00000106966619	-2.82874120681015
I	-0.00000492025138	-0.00000180830719	-5.54580026402201
F	0.00000777005579	0.00000106530698	0.00000047973489

4.5 XYZ files optimized with PBE0-D4/def2-QZVPPD (Orca 6.0.1):

Cl^-

Final single point energy: –460.142336086357 Eh

Total enthalpy: –460.13997561 Eh

Gibbs free energy: –460.15737823 Eh

Cl_2

Zero point energy: 0.00131024 Eh

Final single point energy: –920.118825452423 Eh

Total enthalpy: –920.11403636 Eh

Final Gibbs free energy: –920.13997261 Eh

Cl	-7.18344716247306	2.52812000000000	0.00000000000000
Cl	-5.20441283752693	2.52812000000000	0.00000000000000

$[Cl_3]^-$

Zero point energy: 0.00193785 Eh

Final single point energy: –1380.307885037287 Eh

Total enthalpy: –1380.30046317 Eh

Final Gibbs free energy: –1380.33225847 Eh

Cl	-5.59349818818488	1.65280120409317	0.00000003250249
Cl	-3.30037329505523	1.60279039050706	-0.00000007029296
Cl	-1.00724851675987	1.55277840539977	0.00000003779047

$[Cl(Cl_2)_2]^-$

Zero point energy: 0.00384941 Eh

Final single point energy: –2300.446941994515 Eh

Total enthalpy: –2300.43394238 Eh

Final Gibbs free energy: –2300.47817351 Eh

Cl	-0.00002914263569	1.72537676165102	0.00000010365191
Cl	-0.00000153184384	0.30267608024813	2.03502154768109
Cl	0.00002111166335	-0.87522442787381	3.81294294423941
Cl	-0.00000157998274	0.30267611511763	-2.03502157315126
Cl	0.00002114279892	-0.87522452914296	-3.81294302242113

$[Cl(Cl_2)_3]^-$

Zero point energy: 0.00573210 Eh

Final single point energy: –3220.580376920217 Eh

Total enthalpy: –3220.56124734 Eh

Final Gibbs free energy: –3220.61759671 Eh

Cl	-1.74253404459657	1.63146279672674	0.19292285245733
Cl	-3.13599361616371	2.95179197178978	-0.59996246456244

Cl	-0.53502682535882	-2.33555433952996	0.19442959392281
Cl	-0.95732120964810	-4.20871638895600	-0.59751989571884
Cl	2.27305401088673	0.69920042706054	0.19161207428728
Cl	4.10509246950014	1.26937208520286	-0.60266990168089
Cl	-0.00727078461969	-0.00755655229395	1.22118774329477

$[Cl(Cl_2)_4]^-$

Zero point energy: 0.00740052 Eh

Final single point energy: –4140.711043737140 Eh

Total enthalpy: –4140.68578631 Eh

Final Gibbs free energy: –4140.75226485 Eh

Cl	-1.54450806485461	-1.54450708172910	1.54452355175677
Cl	-2.72841819401053	-2.72840649554097	2.72843114740049
Cl	1.54452057309348	1.54452416381241	1.54454137426361
Cl	2.72841061083022	2.72842632738961	2.72846661295228
Cl	1.54451707209685	-1.54452319939147	-1.54453739473529
Cl	2.72840373748129	-2.72843407209939	-2.72845682563883
Cl	-1.54451498393669	1.54451123759782	-1.54452828676321
Cl	-2.72842886268118	2.72840267526663	-2.72843986775816
Cl	0.00001811198118	0.00000644469446	-0.00000031147766

$[Cl(Cl_2)_5]^-$

Zero point energy: 0.00929395 Eh

Final single point energy: –5060.838882098932 Eh

Total enthalpy: –5060.80738625 Eh

Final Gibbs free energy: –5060.88543786 Eh

Cl	0.01686522927107	-0.00001239242091	0.00002216962927
Cl	0.02456308760130	-0.00002772909829	2.78662412427122
Cl	0.02965718743792	-0.00003777971937	4.81086403171781
Cl	0.02456928270873	-0.00001825107110	-2.78658870086928
Cl	0.02966980456873	-0.00002666690581	-4.81082703879596
Cl	-1.39098089218110	2.34524835258469	-0.00000746744807
Cl	-2.44339403693731	4.08730441383714	-0.00002983279361
Cl	2.75433685764517	0.00003494828638	-0.00000919792704
Cl	4.78920851166393	0.00007388204748	-0.00003515793999
Cl	-1.39102501158484	-2.34525130876301	0.00000058848652

Cl	-2.44347002219362	-4.08728746877721	-0.00001351833082

$[Cl(Cl_2)_6]^-$

Zero point energy: 0.01098058 Eh

Final single point energy: –5980.966911524648 Eh

Total enthalpy: –5980.92921551 Eh

Final Gibbs free energy: –5981.01450920 Eh

Cl	-0.00001258326990	0.00000665691721	-2.81088050658011
Cl	-0.00002059314109	0.00001219747704	-4.83021233023765
Cl	0.00001201361874	2.81090000265108	0.00000699384476
Cl	0.00002268495935	4.83023050500880	0.00001014279644
Cl	-0.00001393063806	0.00000951074776	2.81088243064122
Cl	-0.00002190552258	0.00001780795538	4.83021436923619
Cl	0.00001490614124	-2.81090166914805	0.00000322588110
Cl	0.00002981137797	-4.83023212496938	0.00000506191256
Cl	2.81089742123066	-0.00000949731790	-0.00000585031219
Cl	4.83022775950960	-0.00001676963339	-0.00001074787201
Cl	-2.81090105643110	-0.00000649014348	-0.00000453056427
Cl	-4.83023167715919	-0.00000971045297	-0.00000941979316
Cl	-0.00000285067567	-0.00000041909211	0.00000116104712

F^-

Final single point energy: –99.800595577683 Eh

Total enthalpy: –99.7982351 Eh

Gibbs free energy: –99.81475414 Eh

$[F(Cl_2)]^-$

Zero point energy: 0.00214794 Eh

Final single point energy: –1019.989641651708 Eh

Total enthalpy: –1019.98247643 Eh

Final Gibbs free energy: –1020.00589094 Eh

F	-5.29958205555531	1.64636696853622	0.00000001528104
Cl	-3.44169962320431	1.60590367042270	-0.00000003056584
Cl	-1.15983832124034	1.55609936104109	0.00000001528480

$[F(Cl_2)_2]^-$

Zero point energy: 0.00469434 Eh

Final single point energy: –1940.130439075333 Eh

Total enthalpy: –1940.11711482 Eh

Final Gibbs free energy: –1940.15931583 Eh

F	-0.00002063395588	1.29137789356289	-0.00000023924620
Cl	-0.00000075414219	0.29890964602620	1.83688340782169
Cl	0.00001608390475	-0.65445869559436	3.71260095033101
Cl	-0.00000080488645	0.29890986877431	-1.83688318115054
Cl	0.00001610907979	-0.65445871276903	-3.71260093775599

$[F(Cl_2)_3]^-$

Zero point energy: 0.00667654 Eh

Final single point energy: –2860.266077849013 Eh

Total enthalpy: –2860.24658582 Eh

Final Gibbs free energy: –2860.30086862 Eh

Cl	1.81347481480873	1.26492513488470	0.00001513882048
Cl	3.50046178024216	2.43964627843173	-0.00004775052552
Cl	0.18890744129048	-2.20147837544069	0.00001455397360
Cl	0.36473368711896	-4.24952932892034	-0.00004875685601
Cl	-2.00256869644466	0.93741369524412	0.00001647448466
Cl	-3.86486148452627	1.80784992010419	-0.00004938550286
F	-0.00014754248939	0.00117267469631	0.00008874160565

$[F(Cl_2)_4]^-$

Zero point energy: 0.00867189 Eh

Final single point energy: –3780.398585921836 Eh

Total enthalpy: –3780.37287826 Eh

Final Gibbs free energy: –3780.43495224 Eh

Cl	-1.33122159329816	-1.33121874749095	-1.33122003621852
Cl	-2.50460130445873	-2.50459351785246	-2.50459707463113
Cl	1.33121982296006	1.33121723915961	-1.33121668540683
Cl	2.50459737914287	2.50459497261600	-2.50459308379239
Cl	-1.33122045900712	1.33121831615833	1.33121785163191
Cl	-2.50459803087901	2.50459629377167	2.50459346731127
Cl	1.33122237596780	-1.33121867801651	1.33121931357538
Cl	2.50460165457894	-2.50459341511018	2.50459642885875

F	0.00000015599336	-0.00000246223550	-0.00000018032844

$[F(Cl_2)_5]^-$

Zero point energy: 0.01027607 Eh

Final single point energy: –4700.524349436517 Eh

Total enthalpy: –4700.49247048 Eh

Final Gibbs free energy: –4700.56676035 Eh

F	0.01108072632948	0.00000375976113	0.00000633921367
Cl	0.01685101475254	0.00001129855471	-2.52357456948342
Cl	0.02116164100344	0.00002466928881	-4.52998807955362
Cl	0.01685272644356	0.00001005382045	2.52358136675826
Cl	0.02116142335067	0.00002130738145	4.52999533215817
Cl	-1.19921322433175	-2.03549175881753	0.00000103445613
Cl	-2.23668708166164	-3.77265010167927	-0.00000535517395
Cl	2.38076390816326	-0.00001691924544	0.00000035664897
Cl	4.40398068097681	-0.00003580815063	-0.00000664847979
Cl	-1.19923158711062	2.03548697302345	0.00000011446320
Cl	-2.23672022891575	3.77263652606289	-0.00000989100761

$[F(Cl_2)_6]^-$

Zero point energy: 0.01215172 Eh

Final single point energy: –5620.652340801819 Eh

Total enthalpy: –5620.61425186 Eh

Final Gibbs free energy: –5620.69494227 Eh

Cl	-0.00000168853406	0.00000080308178	-2.50326700553617
Cl	-0.00000508306280	0.00000067593280	-4.51054709341899
Cl	0.00000080520084	2.50326225911828	-0.00000329902927
Cl	0.00000028300843	4.51054316557930	-0.00000784591913
Cl	0.00000086483232	-0.00000064673656	2.50327051766949
Cl	-0.00000113222198	-0.00000208389090	4.51055082235571
Cl	-0.00000004539674	-2.50326260895171	-0.00000246571090
Cl	-0.00000076150180	-4.51054354478652	-0.00000474621991
Cl	2.50326874948843	0.00000060276566	0.00000288941018
Cl	4.51054936306921	0.00000109819211	0.00000535299005
Cl	-2.50326588939345	0.00000032242056	0.00000085690529
Cl	-4.51054675459776	-0.00000026212543	0.00000111443076

F	0.00000128910936	0.00000021940061	0.00000090207289

Br^-

Final single point energy: –2573.991511804200 Eh

Total enthalpy: –2573.98915132 Eh

Gibbs free energy: –2574.00770476 Eh

Br_2

Zero point energy: 0.00076464 Eh

Final single point energy: –5147.815052495536 Eh

Total enthalpy: –5147.81060572 Eh

Final Gibbs free energy: –5147.83907810 Eh

Br	-7.33312670716125	2.52812000000000	0.00000000000000
Br	-5.05473329283873	2.52812000000000	0.00000000000000

$[Br_3]^-$

Zero point energy: 0.00122675 Eh

Final single point energy: –7721.860697750799 Eh

Total enthalpy: –7721.8534707 Eh

Final Gibbs free energy: –7721.88876027 Eh

Br	-5.86596169176416	1.65881718911647	0.00000004724678
Br	-3.30037342497480	1.60279036290160	-0.00000008299704
Br	-0.73478488326102	1.54676244798192	0.00000003575022

$[Br(Br_2)_2]^-$

Zero point energy: 0.00239617 Eh

Final single point energy: –12869.702477832610 Eh

Total enthalpy: –12869.68991774 Eh

Final Gibbs free energy: –12869.73938958 Eh

Br	-0.00003126552015	1.86093033899431	0.00000008743017
Br	-0.00000186518955	0.33559734989046	2.22105313648293
Br	0.00002251888324	-0.97592272368145	4.28221349994059
Br	-0.00000197838797	0.33559808581422	-2.22105341021902
Br	0.00002259021443	-0.97592305101756	-4.28221331363468

$[Br(Br_2)_3]^-$

Zero point energy: 0.00357944 Eh

Final single point energy: –18017.535706094011 Eh

Total enthalpy: –18017.51730407 Eh

Final Gibbs free energy: –18017.57992524 Eh

Br	-1.37977230509191	-2.15278470270891	-0.22632556107697
Br	-2.56863831329804	-4.01793414796948	0.67315229243186
Br	2.56239900423045	-0.12753276791948	-0.22758371530529
Br	4.77144082565417	-0.24662428997562	0.67059483026590
Br	-1.18208801017243	2.28663712849748	-0.22822510013405
Br	-2.20385369321183	4.24941514309944	0.66823201307461
Br	0.00051249288959	0.00882363697660	-1.32984476025607

$[Br(Br_2)_4]^-$

Zero point energy: 0.00471004 Eh

Final single point energy: –23165.364708165362 Eh

Total enthalpy: –23165.34044783 Eh

Final Gibbs free energy: –23165.41428422 Eh

Br	-1.64986777186159	-1.64985827665370	-1.64984155328910
Br	-3.01246113632737	-3.01243145704766	-3.01241600918335
Br	1.64987739083526	-1.64986812669877	1.64984760673286
Br	3.01247915525300	-3.01245034306809	3.01240466255438
Br	1.64986162413548	1.64985407320510	-1.64983417151940
Br	3.01244675976595	3.01244139464400	-3.01240330051805
Br	-1.64987079075948	1.64986254177329	1.64984054671544
Br	-3.01246542390370	3.01245944861703	3.01239018962691
Br	0.00000019286244	-0.00000925477119	0.00001202888031

$[Br(Br_2)_5]^-$

Zero point energy: 0.00563551 Eh

Final single point energy: –28313.189561725991 Eh

Total enthalpy: –28313.15946211 Eh

Final Gibbs free energy: –28313.24602774 Eh

Br	-1.46565234203722	-2.51714191601879	-0.00000372685864
Br	-2.64855821202362	-4.53941519169902	-0.00000496186302
Br	-1.46565576663073	2.51714509299948	0.00000109595888
Br	-2.64856377903217	4.53941730530247	0.00000508098012

Br	2.91955503360699	0.00000026479389	0.00000465635754
Br	5.26232993392247	-0.00000442993661	0.00001337910487
Br	0.00881120211549	-0.00000127427005	-2.96846992286106
Br	0.01240165280778	-0.00000681798689	-5.29810800465800
Br	0.00881647465518	0.00000210546954	2.96846418701019
Br	0.01241447939521	0.00000213945382	5.29810182916417
Br	0.00410132222061	0.00000272189215	-0.00000361233507

$[Br(Br_2)_6]^-$

Zero point energy: 0.00658994 Eh

Final single point energy: –33461.014741049476 Eh

Total enthalpy: –33460.97878752 Eh

Final Gibbs free energy: –33461.07560184 Eh

Br	-0.00001464214263	-0.00000519788484	2.99102606260295
Br	-0.00003371480185	-0.00001190667656	5.31527714499905
Br	-2.99100978430526	0.00000154743855	0.00002878260277
Br	-5.31525934952077	0.00000133898544	0.00004213631621
Br	-0.00000297879314	-0.00000145412918	-2.99100255582225
Br	-0.00001146835407	-0.00000318462225	-5.31525302707008
Br	2.99102923006953	0.00000464485498	0.00001815041659
Br	5.31527972050717	0.00001077288964	0.00001931182686
Br	0.00000220563891	-2.99101757134343	-0.00002430084884
Br	-0.00000374674634	-5.31526792904982	-0.00005460708223
Br	0.00000792494411	2.99101924790822	-0.00002826325367
Br	0.00000421157291	5.31526951921716	-0.00006320611891
Br	0.00001239193143	0.00000017241210	0.00001437143157

$[F(Br_2)]^-$

Zero point energy: 0.00210547 Eh

Final single point energy: –5247.699415656409 Eh

Total enthalpy: –5247.69187699 Eh

Final Gibbs free energy: –5247.72454288 Eh

F	2.88982552229866	0.00006278291793	0.00000003876315
Br	0.93188371797675	0.00002785493933	-0.00000007752631
Br	-1.62755925635241	-0.00001668283626	0.00000003876316

$[F(Br_2)_2]^-$

Zero point energy: 0.00342720 Eh

Final single point energy: –10395.542254361273 Eh

Total enthalpy: –10395.52939261 Eh

Final Gibbs free energy: –10395.57591968 Eh

F	0.00000015774205	0.00000000774038	-1.36039788164482
Br	-1.94486647300131	0.00000005039058	-0.41331762792963
Br	-4.13210762926567	-0.00000002358005	0.57711471688125
Br	1.94486631511324	-0.00000006553266	-0.41331767134147
Br	4.13210762941169	0.00000003098175	0.57711470122667

$[F(Br_2)_3]^-$

Zero point energy: 0.00462755 Eh

Final single point energy: –15543.376766766558 Eh

Total enthalpy: –15543.35806241 Eh

Final Gibbs free energy: –15543.41814016 Eh

Br	-4.63869791440574	0.16242588943755	-0.00000316621037
Br	-2.28661542186126	0.07803262830158	-0.00000855101187
F	0.00125004643312	-0.00265951760273	-0.00001388333805
Br	1.20940490477266	1.94215239275715	-0.00000858181758
Br	1.07825818502276	-2.02255662377443	-0.00000871886436
Br	2.45049197025191	3.94180628706971	-0.00000314069744
Br	2.18590822978656	-4.09920105618983	-0.00000304581532

$[F(Br_2)_4]^-$

Zero point energy: 0.00582230 Eh

Final single point energy: –20691.206728839039 Eh

Total enthalpy: –20691.18216097 Eh

Final Gibbs free energy: –20691.25106630 Eh

Br	-1.38150086181154	-1.38150140672045	1.38150080591185
Br	-2.72681090925668	-2.72681186958938	2.72680976821191
Br	1.38150040241780	1.38150109866359	1.38150005479657
Br	2.72681039301246	2.72681195878222	2.72680862614265
Br	1.38150027355028	-1.38150110027896	-1.38150002212210
Br	2.72681036976130	-2.72681041369211	-2.72681013822478
Br	-1.38150023008524	1.38150068677256	-1.38150000814525

Br	-2.72680912285213	2.72681158361291	-2.72680964999647
F	-0.00000031473625	-0.00000053755039	0.00000056123862

$[F(Br_2)_5]^-$

Zero point energy: 0.00660825 Eh

Final single point energy: –25839.028060343950 Eh

Total enthalpy: –25838.99768667 Eh

Final Gibbs free energy: –25839.08059160 Eh

Br	1.21710913644264	2.10314208338959	-0.00000034395111
Br	2.38323040683500	4.11461540089075	0.00000181346489
Br	-2.43188400044210	-0.00000045764469	-0.00000049661819
Br	-4.75708682840471	-0.00000083853886	0.00000115125241
Br	1.21710900441549	-2.10314268318551	-0.00000032174323
Br	2.38322987267197	-4.11461625966132	0.00000190841932
Br	-0.00224269992221	-0.00000057421113	2.71064046187722
Br	-0.00292931751843	0.00000205238480	5.01095602708291
Br	-0.00224268927503	-0.00000034839888	-2.71064117074574
Br	-0.00293008374626	0.00000202223830	-5.01095694747165
F	-0.00136280105437	-0.00000039726307	-0.00000208156681

$[F(Br_2)_6]^-$

Zero point energy: 0.00757106 Eh

Final single point energy: –30986.852546503229 Eh

Total enthalpy: –30986.81635441 Eh

Final Gibbs free energy: –30986.90677444 Eh

Br	0.00000042396567	2.61815929503238	0.00000028538212
Br	0.00000061537875	4.92344631488570	0.00000054978147
Br	2.61815917876544	-0.00000011892557	-0.00000012656300
Br	4.92344621015098	0.00000116506571	-0.00000031731002
Br	0.00000011152420	-2.61815956605672	-0.00000006245749
Br	0.00000051929848	-4.92344666223564	-0.00000015985064
Br	-2.61815898188308	0.00000003465752	0.00000004391108
Br	-4.92344603834597	0.00000082116557	0.00000013342441
Br	-0.00000023444756	-0.00000032408562	-2.61815843280468
Br	-0.00000046772483	-0.00000043771803	-4.92344552749237
Br	-0.00000044551746	-0.00000039270201	2.61815825356631

Br	-0.00000089687157	0.00000049337524	4.92344534299021
F	0.00000000570694	-0.00000062245854	0.00000001742260

I^-

Final single point energy: –297.813008898567 Eh

Total enthalpy: –297.81064842 Eh

Final Gibbs free energy: –297.82985706 Eh

I_2

Zero point energy: 0.00051903 Eh

Final single point energy: –595.466071292077 Eh

Total enthalpy: –595.46172918 Eh

Final Gibbs free energy: –595.49189459 Eh

I	-1.32484117576109	0.00000000000000	0.00000000000000
I	1.32484117576109	0.00000000000000	0.00000000000000

$[I_3]^-$

Zero point energy: 0.00085394 Eh

Final single point energy: –893.335679528339 Eh

Total enthalpy: –893.32852435 Eh

Final Gibbs free energy: –893.36590765 Eh

I	-2.92370382939724	0.00000020280516	-0.00000000200044
I	0.00000001592843	-0.00000040560934	0.00000000400088
I	2.92370381346881	0.00000020280518	-0.00000000200044

$[I(I_2)_2]^-$

Zero point energy: 0.00170599 Eh

Final single point energy: –1488.832604030092 Eh

Total enthalpy: –1488.82017908 Eh

Final Gibbs free energy: –1488.87295991 Eh

I	-0.00000004729142	0.00000004678057	-2.00795644766991
I	-2.47183688676598	-0.00000007067241	-0.26588410073334
I	-4.83559450407006	0.00000003714042	1.26986225778750
I	2.47183690620911	-0.00000002917697	-0.26588388049389
I	4.83559453191835	0.00000001592840	1.26986217110864

$[I(I_2)_3]^-$

Zero point energy: 0.00255307 Eh

Final single point energy: –2084.319943920383 Eh

Total enthalpy: –2084.30176722 Eh

Final Gibbs free energy: –2084.36843365 Eh

I	-2.30517649686719	-1.59990181034891	0.28592802116710
I	-4.37883986323917	-3.06042175564447	-0.82125537453185
I	2.53726606453060	-1.20914106076039	0.28899406324603
I	4.81569449376033	-2.32055548411528	-0.82140689919334
I	-0.23168057360763	2.82232500477966	0.29028517549690
I	-0.43816402106542	5.35242585473785	-0.81326775077046
I	0.00090039648747	0.01526925135053	1.59072276458662

$[I(I_2)_4]^-$

Zero point energy: 0.00331060 Eh

Final single point energy: –2679.801958148306 Eh

Total enthalpy: –2679.77804044 Eh

Final Gibbs free energy: –2679.85612046 Eh

I	-1.82213531416380	1.82213509785772	1.82213513797592
I	-3.40443067143054	3.40442696179644	3.40442932123870
I	-1.82213446796446	-1.82213420640238	-1.82213439979698
I	-3.40442861937105	-3.40442883999644	-3.40442688678164
I	1.82213599593346	1.82213628949701	-1.82213629679742
I	3.40442923472799	3.40442937610598	-3.40443142852968
I	1.82213524106098	-1.82213548571834	1.82213552561234
I	3.40442698656636	-3.40443106015847	3.40442951368306
I	0.00000161464106	0.00000186701849	-0.00000048660431

$[I(I_2)_5]^-$

Zero point energy: 0.00397921 Eh

Final single point energy: –3275.280034388510 Eh

Total enthalpy: –3275.25036287 Eh

Final Gibbs free energy: –3275.34315324 Eh

I	-1.55529211896921	-2.81829264449141	0.00000026178342
I	-2.88185598695154	-5.19495634525075	0.00000090691694
I	-1.67224647955461	2.72288679662771	-0.00000003508658

I	-3.08975272419965	5.04692665413234	-0.00000065339144
I	3.21197423465743	0.11142192813455	0.00000087086946
I	5.93143818236639	0.22106532910347	0.00000179132032
I	0.01056476418617	-0.01747052196779	-3.25364040860258
I	0.01524076988280	-0.02061661639318	-5.96173594563945
I	0.01056559810623	-0.01747154580408	3.25363932390697
I	0.01524231488652	-0.02061737472736	5.96173469917441
I	0.00412144558949	-0.01287565936349	-0.00000081125146

$[I(I_2)_6]^-$

Zero point energy: 0.00462062 Eh

Final single point energy: –3870.758285791239 Eh

Total enthalpy: –3870.72285869 Eh

Final Gibbs free energy: –3870.82598324 Eh

I	-3.27312947833046	0.00000014036829	-0.00000063114863
I	-5.97501743715975	0.00000065595580	-0.00000147831108
I	0.00000043395209	-0.00000045666884	3.27312923561376
I	0.00000097683029	-0.00000069725422	5.97501724348990
I	3.27312924040179	0.00000020389963	-0.00000015486131
I	5.97501716669108	0.00000098020558	-0.00000032439938
I	-0.00000000454946	-0.00000001297968	-3.27312907683616
I	-0.00000019788673	0.00000030617016	-5.97501700107737
I	-0.00000004791197	3.27312901961316	0.00000028368169
I	0.00000002366415	5.97501699416726	-0.00000003953529
I	-0.00000017944440	-3.27312937749441	0.00000068517675
I	-0.00000026334766	-5.97501743754222	0.00000098701021
I	-0.00000023290896	-0.00000031844050	0.00000027119692

$[F(I_2)]^-$

Zero point energy: 0.00184131 Eh

Final single point energy: –695.362709177737 Eh

Total enthalpy: –695.35523295 Eh

Final Gibbs free energy: –695.38960440 Eh

F	3.28566611991620	-0.00011841990631	0.00000002302519
I	1.21324702808774	-0.00005628001609	-0.00000004605022
I	-1.70513191195994	0.00003178667540	0.00000002302503

$[F(I_2)_2]^-$

Zero point energy: 0.00279016 Eh

Final single point energy: –1290.861180887618

Total enthalpy: –1290.84848672 Eh

Final Gibbs free energy: –1290.89767479 Eh

F	0.00000054323624	0.00000002490068	1.34801600924027
I	2.09363682468159	-0.00000010439418	0.46068183409728
I	4.66857800929542	0.00000004719270	-0.56163355807650
I	-2.09363731264855	0.00000005788089	0.46068137661778
I	-4.66857806456471	-0.00000002558008	-0.56163346828382

$[F(I_2)_3]^-$

Zero point energy: 0.00362049 Eh

Final single point energy: –1886.348646311275 Eh

Total enthalpy: –1886.3302188 Eh

Final Gibbs free energy: –1886.39375312 Eh

I	-2.28712852446542	-0.74492650741586	-0.00000444152485
I	-4.87554176825078	-1.59054208093041	0.00000386735229
I	1.79608486482124	-1.61654497712061	-0.00000434220004
I	3.81399387056935	-3.44512231369083	0.00000381042831
I	0.49884900636923	2.36368301155895	-0.00000434242967
I	1.04613379552582	5.03140785006799	0.00000381551487
F	0.00760875543157	0.00204501753176	-0.00001124954791

$[F(I_2)_4]^-$

Zero point energy: 0.00442335 Eh

Final single point energy: –2481.830735190911 Eh

Total enthalpy: –2481.80656614 Eh

Final Gibbs free energy: –2481.87964464 Eh

I	1.45983204139269	1.45983278201698	-1.45983321259308
I	3.01824275752048	3.01824494610525	-3.01824529557583
I	-1.45983254185998	1.45983364714346	1.45983431235979
I	-3.01824234980870	3.01824653264688	3.01824647815016
I	-1.45983279564848	-1.45983403802893	-1.45983482823470
I	-3.01824316993797	-3.01824548342052	-3.01824746199482
I	1.45983250077590	-1.45983332927777	1.45983390878736

I	3.01824398960760	-3.01824433264112	3.01824612264651
F	-0.00000043060455	-0.00000072310723	-0.00000002210838

$[F(I_2)_6]^-$

Zero point energy: 0.00533051 Eh

Final single point energy: –3672.779223212563 Eh

Total enthalpy: –3672.74365291 Eh

Final Gibbs free energy: –3672.84205224 Eh

I	2.78855063822337	-0.00000191875038	0.00000077987195
I	5.46482082675131	0.00000153166846	-0.00000042225999
I	0.00000342220082	2.78855260061124	-0.00000014644685
I	-0.00000695164593	5.46482202581172	-0.00000089125306
I	-2.78854854419283	0.00000038836842	-0.00000008894460
I	-5.46481803048342	0.00000141485777	-0.00000172958842
I	0.00000158252956	-2.78855283997550	0.00000131568321
I	-0.00000495245964	-5.46482263044294	-0.00000145455648
I	0.00000261918951	-0.00000001198490	2.78854839396462
I	-0.00000461121271	0.00000107996806	5.46481837052074
I	0.00000262482302	-0.00000119069583	-2.78854783215796
I	-0.00000636473671	0.00000186420020	-5.46481783779637
F	0.00000774101360	-0.00000231363631	0.00000154296324

4.6 XYZ files optimized with M062X/def2-QZVPPD (Orca 6.0.1):

F^-

Final single point energy: –99.860586611118 Eh

Total enthalpy: –99.85822613 Eh

Final Gibbs free energy: –99.87474518 Eh

I_2

Zero point energy: 0.00052962 Eh

Final single point energy: –595.280996788660 Eh

Total enthalpy: –595.27665091 Eh

Final Gibbs free energy: –595.30679722 Eh

I	-1.32347645991154	0.00000000000000	0.00000000000000
I	1.32347645991154	0.00000000000000	0.00000000000000

$[F(I_2)]^-$

Zero point energy: 0.00191356 Eh

Final single point energy: –695.235568655258 Eh

Total enthalpy: –695.22805184 Eh

Final Gibbs free energy: –695.26236648 Eh

F	-3.27218209546141	-0.00035833522919	0.00000004052567
I	-1.22098275599347	-0.00017449815148	-0.00000008106264
I	1.71086496637388	0.00009873120667	0.00000004053697

$[F(I_2)_2]^-$

Zero point energy: 0.00269651 Eh

Final single point energy: –1290.545029173717 Eh

Total enthalpy: –1290.53236626 Eh

Final Gibbs free energy: –1290.58201792 Eh

F	-0.00000000164387	-0.00000002037074	1.34803344113867
I	-0.00000000804253	-2.09372047735478	0.46757364570442
I	0.00000000360074	-4.66119347398380	-0.56128491163225
I	0.00000001107428	2.09372049728959	0.46757360949472
I	-0.00000000498862	4.66119347441973	-0.56128489952155

$[F(I_2)_3]^-$

Zero point energy: 0.00352834 Eh

Final single point energy: –1885.846429005397 Eh

Total enthalpy: –1885.82802982 Eh

Final Gibbs free energy: –1885.89200676 Eh

I	-1.52969104321730	1.87738540761619	0.00000278798816
I	-3.23865077915464	3.99017390106757	-0.00000183215430
I	-0.86280592733347	-2.27254863351930	0.00000267264384
I	-1.81766244310870	-4.81656235985947	-0.00000150478723
I	2.38750489905104	0.38853112234368	0.00000218415663
I	5.06648955021022	0.83958030972647	-0.00000156088527
F	-0.00518425644715	-0.00655974737514	0.00000606704217

$[F(I_2)_4]^-$

Zero point energy: 0.00450812 Eh

Final single point energy: –2481.144267334425 Eh

Total enthalpy: –2481.12008072 Eh

Final Gibbs free energy: –2481.19521273 Eh

I	-0.00112493716714	0.01711379494930	0.02419623035557
I	2.69187511108081	0.08389621678505	0.08743542816435
I	-3.39902513887700	2.35522879556165	-0.02700926066013
I	-4.30215874373848	4.89366424050211	-0.02749386097038
I	-3.35210835525880	-1.22833434651998	-2.10725597216139
I	-4.22687570523145	-2.49701637052128	-4.31807573139886
I	-3.39790650976444	-1.23799647466089	2.03505763851153
I	-4.31215593382603	-2.51523860544835	4.22434101604901
F	-2.53731059766348	-0.03429186498393	-0.02603341889539

$[F(I_2)_5]^-$

Zero point energy: 0.00511689 Eh

Final single point energy: –3076.431874744756 Eh

Total enthalpy: –3076.40195528 Eh

Final Gibbs free energy: –3076.49026922 Eh

I	0.01029472593420	-0.00828443805000	-0.00086930028761
I	2.70084625558116	-0.02651560391128	0.00176160583281
I	-3.88297344037589	2.22455904205350	-0.00071610497591
I	-5.25565341453172	4.53856676615286	0.00102564810140
I	-3.87283053878450	-2.21787237397805	-0.00447240664580
I	-5.23600159585483	-4.53758982946137	-0.00602725512447
I	-2.56531950525262	0.00533384813461	2.92110150864803
I	-2.55889476777270	0.00578331521146	5.58951540762801
I	-2.56282398673928	0.01049086232169	-2.92500283467564
I	-2.55234344509400	0.01864128513337	-5.59343456659421
F	-2.56934651114484	0.00575866547025	-0.00265716503917

$[F(I_2)_6]^-$

Zero point energy: 0.00584808 Eh

Final single point energy: –3671.724896298754 Eh

Total enthalpy: –3671.68923809 Eh

Final Gibbs free energy: –3671.78561603 Eh

I	-0.00000045112425	2.79980820558832	-0.00000004871046
I	0.00000105428134	5.47278725636722	0.00000077852685

I	2.79980699361334	0.00000111879030	0.00000112922792
I	5.47278589227332	-0.00000264473994	0.00000059213488
I	0.00000027704264	-2.79980597420372	-0.00000057254124
I	0.00000016208410	-5.47278478831274	-0.00000009904342
I	-2.79980721252928	0.00000033047382	-0.00000149883923
I	-5.47278616232950	-0.00000276177818	0.00000191243856
I	0.00000120664457	0.00000093433410	-2.79980728601691
I	-0.00000064817664	-0.00000307347430	-5.47278625946125
I	-0.00000127107699	0.00000056499682	2.79980666513187
I	0.00000064189998	-0.00000173935564	5.47278556950648
F	-0.00000048260265	0.00000257131395	-0.00000088235405

4.7 XYZ files optimized with B3LYP-D3(*BJ*)/def2-QZVPPD (Orca 5.0.3):

F_2

Zero point energy: 0.00238193 Eh

Final single point energy: –199.542612993895 Eh

Final Gibbs free energy: –199.55984801 Eh

F -6.89214334552830 2.52812000000000 0.00000000000000

F -5.49571665447169 2.52812000000000 0.00000000000000

ClF

Zero point energy: 0.00178328 Eh

Final single point energy: –559.955867441888 Eh

Final Gibbs free energy: –559.97543322 Eh

F -7.01363090795132 2.52812000000000 0.00000000000000

Cl -5.37422909204868 2.52812000000000 0.00000000000000

BrF

Zero point energy: 0.00152587 Eh

Final single point energy: –2673.897459858785 Eh

Final Gibbs free energy: –2673.91849516 Eh

F -7.07995259407534 2.52812000000000 0.00000000000000

Br -5.30790740592465 2.52812000000000 0.00000000000000

IF

Zero point energy: 0.00139466 Eh

Final single point energy: –397.537984397245 Eh

Final Gibbs free energy: –397.55993640 Eh

F 0.00000000000000 0.00000000000000 0.37042616751234

I 0.00000000000000 0.00000000000000 2.29157383248766

ClF_3

Zero point energy: 0.00683653 Eh

Final single point energy: –759.549444422300 Eh

Final Gibbs free energy: –759.57015636 Eh

Cl -3.95748984310503 1.10926538722147 0.02715809817288

F -5.67446199010571 1.19548541747532 0.01740051290382

F -2.25218015631702 0.90061113689098 0.02888849147908

F -4.09407789402191 -0.49852126477465 0.00148116616176

BrF_3

Zero point energy: 0.00596112 Eh

Final single point energy: –2873.524606301375 Eh

Final Gibbs free energy: –2873.54735114 Eh

Br -3.96281866815576 1.15331452250137 0.02100866582807

F -5.79299503289858 1.18858141073912 0.01369329785312

F -2.15178559692002 0.88321481150882 0.02524904665415

F -4.10872334153885 -0.58019179264782 0.00284346061739

IF_3

Zero point energy: 0.00555571 Eh

Final single point energy: –597.219847153554 Eh

Final Gibbs free energy: –597.24393798 Eh

I -3.93263093013734 1.20136275904500 0.01854134047722

F -5.88517184946080 1.17348704354457 0.00987380237855

F -2.01762069163802 0.82331281690184 0.01966382826841

F -4.10216670809316 -0.66507379359169 0.00741226475683

ClF_5

Zero point energy: 0.01319307 Eh

Final single point energy: –959.123413916330 Eh

Final Gibbs free energy: –959.14005767 Eh

Cl -4.11458114611306 0.60506693614932 0.09051645078497

F -5.01911316622395 1.65465727395928 1.05193489649688

F -2.74221242776388 0.96707868410046 1.00103164607661

F -3.25145602933030 -0.59715554225917 -0.71917464121201

F -5.53086165047230 0.08984176343456 -0.66685057027515

F -4.42798720886946 -0.51614825133187 1.20407136011606

BrF_5

Zero point energy: 0.01174550 Eh

Final single point energy: –3073.146906362368 Eh

Final Gibbs free energy: –3073.16616090 Eh

Br	-4.10736984822142	0.63124589720174	0.06421921453282
F	-5.07296754729531	1.71400018815897	1.11172912001888
F	-2.66231822415247	0.98522254245409	1.05775752734093
F	-3.20024698290205	-0.67084800592723	-0.76387560423361
F	-5.61589045693491	0.05636922460513	-0.70797145353520
F	-4.44221899711975	-0.56581827446043	1.25305746277255

IF_5

Zero point energy: 0.01107056 Eh

Final single point energy: –796.920995312647 Eh

Final Gibbs free energy: –796.94204328 Eh

I	-4.09863306328270	0.66473725107982	0.03124045029791
F	-5.13285668954159	1.76677069188408	1.18553152836079
F	-2.58036320566591	0.99227941474739	1.12616412527665
F	-3.14857423108838	-0.75772626427200	-0.79940741807143
F	-5.70790516546417	0.00993947538328	-0.74165964980759
F	-4.45600209722845	-0.61287058882921	1.29941263043020

4.8 Solid-state calculations with the Crystal program:

Periodic density functional theory (DFT) calculations were performed using the B3LYP functional and the CRYSTAL17 program.[53] All DFT structure optimizations made use of dispersion correction according to Grimme's D3 scheme[44,45,47] as implemented in the CRYSTAL17 program. For the periodic calculations, six grid points along each direction were used for k-space sampling. Truncation criteria for 2-electron integrals (thresholds for the overlap of atomic orbitals from pairs of atoms, which determine if Coulomb or exchange integrals are evaluated or not; TOLINTEG, cf. Crystal17 manual) of 10^{-8}, 10^{-8}, 10^{-8}, 10^{-8} and 10^{-16} were chosen. For fluorine, Dunnings cc-pVDZ basis set was used.[54] For bromine, a pseudopotential (PP) and the corresponding basis set was obtained from the Stuttgart-Cologne group.[55] The quasi-relativistic large-core PP from an energy-consistent, multi-electron fit mimicking a chemically inactive $[Ar]3d^{10}$ core. The valence electrons were represented by contracted Gaussian type orbital (CGTO) sets optimized for this PP,[56] removing the most diffuse s-, p- and d-function. However, for technical reasons, the first s-function needed to be partly decontracted, as described previously in the supplementary information of a study of solid Br_2.[57]

4.8.1 Calculated density of states:

The projected density of states (DOS) are based on the optimised structures in CRYSTAL17. Bands with negative energy are occupied, bands with positive energy are unoccupied.

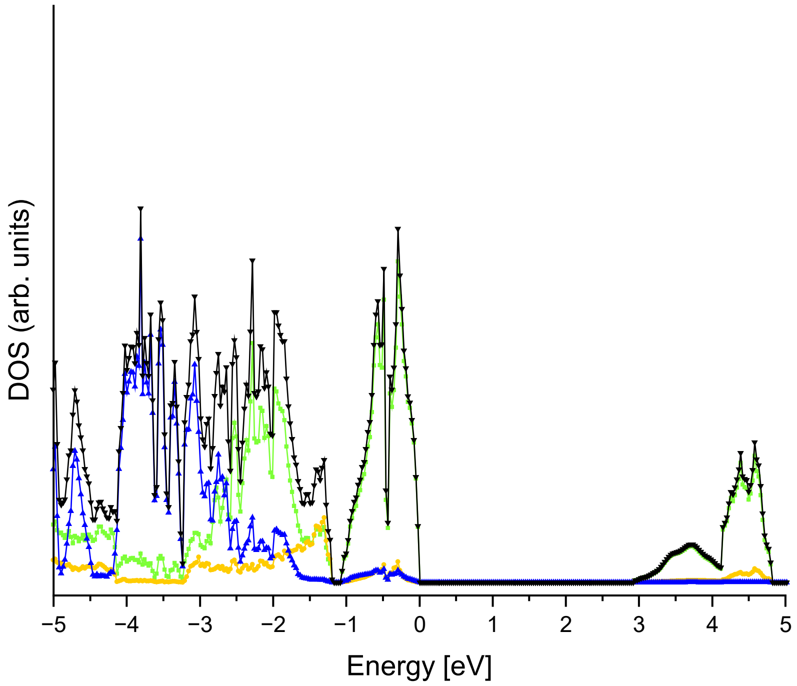

Figure S20: Density of state for the optimized $[NEt_4][F(Cl_2)_3]$. The band gap is 2.93 eV. The DOS for all atoms is represented in black, the DOS for the fluoride anion in yellow, for the chlorine atoms in green and for all atoms of the cation in blue.

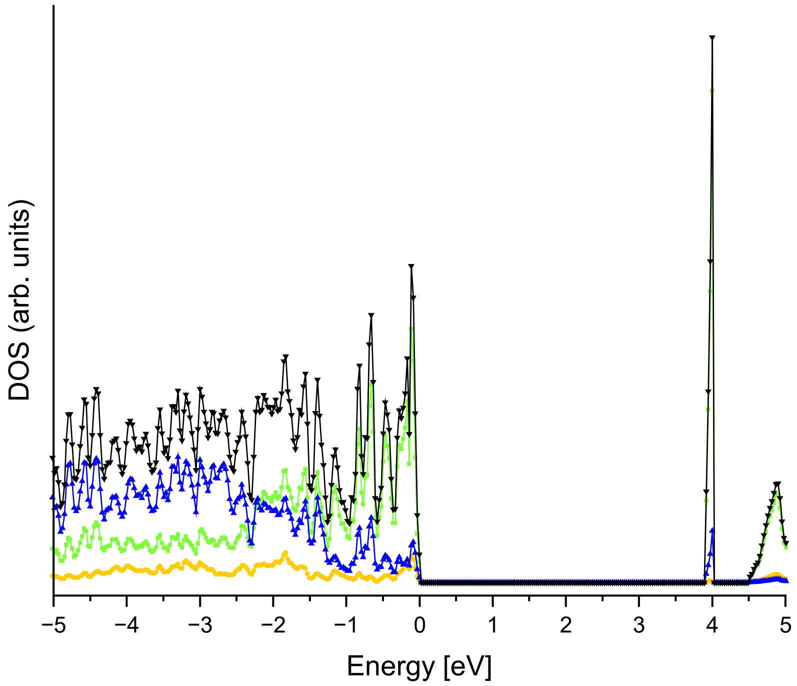

Figure S21: Density of state for the optimized $[NEt_4][F(Cl_2)_3]$ under external pressure. The band gap is 3.89 eV. The DOS for all atoms is represented in black, the DOS for the fluoride anion in yellow, for the chlorine atoms in green and for all atoms of the cation in blue.

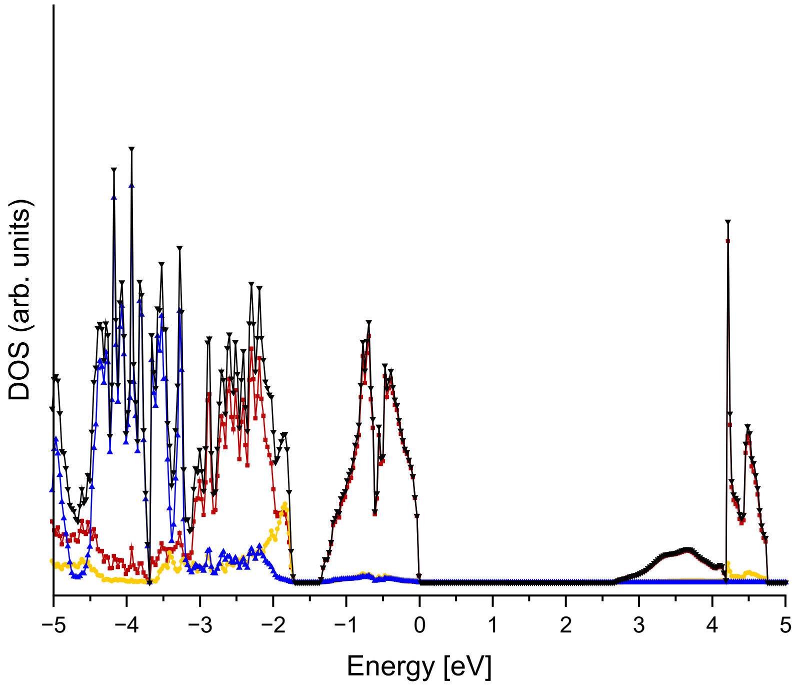

Figure S22: Density of state for the optimized [NEt_4][$F(Br_2)_3$]. The band gap is 2.67 eV. The DOS for all atoms is represented in black, the DOS for the fluoride anion in yellow, for the bromine atoms in red and for all atoms of the cation in blue.

5. Literature:

(1) Sonnenberg, K.; Mann, L.; Redeker, F. A.; Schmidt, B.; Riedel, S. Polyhalogen and Polyinterhalogen Anions from Fluorine to Iodine. *Angew. Chem. Int. Ed.* **2020**, *59*, 5464-5493.
(2) Möbs, M.; Graubner, T.; Karttunen, A. J.; Kraus, F. $[(\mu_3\text{-F})(BrF_5)_3]^-$ – An Unprecedented Molecular Fluoridobromate(V) Anion in $Cs[Br_3F_{16}]$. *Chemistry* **2023**, *29*, e202301876.
(3) Pröhm, P.; Schwarze, N.; Müller, C.; Steinhauer, S.; Beckers, H.; Rupf, S. M.; Riedel, S. Non-classical polyinterhalides of chlorine monofluoride: experimental and theoretical characterization of $[F(ClF)_3]^-$. *Chem. Commun.* **2021**, *57*, 4843-4846.
(4) Ivlev, S. I.; Karttunen, A. J.; Buchner, M. R.; Conrad, M.; Kraus, F. The Interhalogen Cations $[Br_2F_5]^+$ and $[Br_3F_8]^+$. *Angew. Chem. Int. Ed.* **2018**, *57*, 14640-14644.
(5) Ivlev, S.; Woidy, P.; Sobolev, V.; Gerin, I.; Ostvald, R.; Kraus, F. On On Tetrafluorobromates(III): Crystal Structures of the Dibromate $CsBr_2F_7$ and the Monobromate $CsBrF_4$. *Z. Anorg. Allg. Chem.* **2013**, *639*, 2846-2850.
(6) Ivlev, S. I.; Woidy, P.; Zherin, I. I.; Ostvald, R. V.; Kraus, F.; Voytenko, M. Y.; Shagalov, V. V. Crystallographic Studies of Cesium Tetrafluorobromates(III). *Procedia Chem.* **2014**, *11*, 35-42.
(7) Ivlev, S. I.; Karttunen, A. J.; Ostvald, R. V.; Kraus, F. $Br_2F_7^-$ and $Br_3F_{10}^-$: peculiar anions showing μ_2- and μ_3-bridging F-atoms. *Chem. Commun.* **2016**, *52*, 12040-12043.
(8) Bandemehr, J.; Sachs, M.; Ivlev, S. I.; Karttunen, A. J.; Kraus, F. $PbF[Br_2F_7]$, a Fluoridobromate(III) of a p-Block Metal. *Eur. J. Inorg. Chem.* **2019**, *2020*, 64-70.
(9) Schmid, J. R.; Pröhm, P.; Voßnacker, P.; Thiele, G.; Ellwanger, M.; Steinhauer, S.; Riedel, S. Soluble Fluoridobromates as Well-Behaved Strong Fluorination Reagents. *Eur. J. Inorg. Chem.* **2020**, *2020*, 4497-4500.
(10) Mahjoub, A. R.; Hoser, A.; Fuchs, J.; Seppelt, K. The Structure of BrF_6^- and Related-Compounds. *Angew. Chem. Int. Ed.* **1989**, *28*, 1526-1527.
(11) Christe, K. O. Alkali metal fluoride-iodine pentafluoride adducts. *Inorg. Chem.* **1972**, *11* (6), 1215-1219.
(12) Scheibe, B.; Karttunen, A. J.; Müller, U.; Kraus, F. $Cs[Cl_3F_{10}]$: A Propeller-Shaped $[Cl_3F_{10}]^-$ Anion in a Peculiar $A^{[5]}B^{[5]}$ Structure Type. *Angew. Chem. Int. Ed.* **2020**, *59*, 18116-18119.
(13) Bandemehr, J.; Ivlev, S. I.; Karttunen, A. J.; Kraus, F. Preparation of Two Quantum-Chemically Predicted, Isomeric $[Br_4F_{13}]^-$ Anions in the Solid State. *Eur. J. Inorg. Chem.* **2020**, *2020*, 4568-4576.
(14) Koch, B. N.; Graubner, T.; Kraus, F. The Peculiar Molecular $[(IF_5)_6(HF_2)_4]^{4-}$ Anion of the Compound $K_4[(IF_5)_6(HF_2)_4]$. *Z. Anorg. Allg. Chem.* **2024**, *650*, e202400085.
(15) Möbs, M.; Karttunen, A. J.; Kraus, F. Fluoridobromate(V)–Hydrogen Fluoride Cocrystallizates. *Acta Chim. Slov.* **2025**, *72*, 1-9.
(16) Möbs, M.; Graubner, T.; Karttunen, A. J.; Kraus, F. $[Br_4F_{21}]^-$ – a unique molecular tetrahedral interhalogen ion containing a μ_4-bridging fluorine atom surrounded by BrF_5 molecules. *Chem. Sci.* **2024**, *15*, 3273-3278.
(17) Pröhm, P.; Schmid, J. R.; Sonnenberg, K.; Voßnacker, P.; Steinhauer, S.; Schattenberg, C. J.; Müller, R.; Kaupp, M.; Riedel, S. Improved Access to Organo-Soluble Di- and Tetrafluoridochlorate(I)/(III) Salts. *Angew. Chem. Int. Ed.* **2020**, *59*, 16002-16006.
(18) Bondi, A. Van der Waals Volumes and Radii. *J. Phys. Chem.* **1964**, *68*, 441-451.
(19) Voßnacker, P.; Keilhack, T.; Schwarze, N.; Sonnenberg, K.; Seppelt, K.; Malischewski, M.; Riedel, S. From Missing Links to New Records: A Series of Novel Polychlorine Anions. *Eur. J. Inorg. Chem.* **2021**, *2021*, 1034-1040.
(20) Sonnenberg, K.; Pröhm, P.; Schwarze, N.; Müller, C.; Beckers, H.; Riedel, S. Investigation of Large Polychloride Anions: $[Cl_{11}]^-$, $[Cl_{12}]^{2-}$, and $[Cl_{13}]^-$. *Angew. Chem. Int. Ed.* **2018**, *57*, 9136-9140.
(21) Powell, B. M.; Heal, K. M.; Torrie, B. H. The temperature dependence of the crystal structures of the solid halogens, bromine and chlorine. *Mol. Phys.* **1984**, *53*, 929-939.
(22) DesMarteau, D. D.; Grelbig, T.; Hwang, S.-H.; Seppelt, K. CsF·Br2, an Alkali-Metal Halide Intercalation Compound. *Angew. Chem. Int. Ed.* **1990**, *29*, 1448-1449.
(23) Drews, T.; Marx, R.; Seppelt, K. Cesium Fluoride–Bromine Intercalation Compounds. *Chem. Eur. J.* **1996**, *2*, 1303-1307.
(24) Haller, H.; Schröder, J.; Riedel, S. Structural Evidence for Undecabromide $[Br_{11}]^-$. *Angew. Chem. Int. Ed.* **2013**, *52*, 4937-4940.
(25) Cahill, J. E.; Leroi, G. E. Raman Spectra of Solid Chlorine and Bromine. *J. Chem. Phys.* **1969**, *51*, 4514-4519.
(26) Desiraju, G. R.; Ho, P. S.; Kloo, L.; Legon, A. C.; Marquardt, R.; Metrangolo, P.; Politzer, P.; Resnati, G.; Rissanen, K. Definition of the halogen bond (IUPAC Recommendations 2013). *Pure Appl. Chem.* **2013**, *85*, 1711-1713.
(27) Ivlev, S. I.; Buchner, M. R.; Karttunen, A. J.; Kraus, F. Synthesis and characterization of the pyridine—bromine trifluoride (1/1) complex, $[py{\cdot}BrF_3]$. *J. Fluor. Chem.* **2018**, *215*, 17-24.
(28) Poręba, T.; Ernst, M.; Zimmer, D.; Macchi, P.; Casati, N. Pressure-Induced Polymerization and Electrical Conductivity of a Polyiodide. *Angew. Chem. Int. Ed.* **2019**, *58*, 6625-6629.
(29) Krause, L.; Herbst-Irmer, R.; Sheldrick, G. M.; Stalke, D. Comparison of silver and molybdenum microfocus X-ray sources for single-crystal structure determination. *J. Appl. Cryst.* **2015**, *48*, 3-10.
(30) APEX-IV (Version 4.0), Data Reduction and Frame Integration Programfor the CCD Area-Detector System, Bruker AXS Inc.: Madison, Wisconsin (USA), **2021**.
(31) Sheldrick, G. M. SHELXT - integrated space-group and crystal-structure determination. *Acta Cryst.* **2015**, *A71*, 3-8.
(32) Sheldrick, G. M. Crystal structure refinement with SHELXL. *Acta Cryst.* **2015**, *71*, 3-8.
(33) Dolomanov, O. V.; Bourhis, L. J.; Gildea, R. J.; Howard, J. A. K.; Puschmann, H. *OLEX2*: a complete structure solution, refinement and analysis program. *J. Appl. Cryst.* **2009**, *42*, 339-341.

(34) Putz, H.; Brandenburg, K. DIAMOND, Crystal and Molecular Structure Visualization (Version 4.65); Crystal Impact: Bonn (Germany), **2023**.
(35) Pröhm, P.; Berg, W. R.; Rupf, S. M.; Voßnacker, P.; Riedel, S. Investigation of Bis(Perfluoro-*tert*-Butoxy) Halogenates(I/III). *Chem. Eur. J.* **2021**, *27*, 17676-17681.
(36) OPUS (Version 7.5); Bruker Optik GmbH: Ettlingen (Germany), **2014**.
(37) OMNIC (Version 9.7.46); Thermo Fisher Scintific Inc.: Waltham, Massachusetts (USA), **2016**.
(38) ORIGINPRO (Version 9.9.0.220), Data Analysis and Graphing Software; OriginLab Corp.: Northampton, Massachusetts (USA), **2022**.
(39) Bennett, L.; Melchers, B.; Proppe, B., *Freie Universität Berlin,* **2020**, https://doi.org/10.17169/refubium-26754.
(40) Neese, F. The ORCA program system. *WIREs Comput. Mol. Sci.* **2011**, *2*, 73-78.
(41) Neese, F. Software update: The ORCA program system-Version 5.0. *WIREs Comput. Mol. Sci.* **2022**, *12*, e1606.
(42) Neese, F. Software Update: The ORCA Program System—Version 6.0. *WIREs Comput. Mol. Sci.* **2025**, *15*, e70019.
(43) Frisch, M. J.; Trucks, G. W.; Schlegel, H. B.; Scuseria, G. E.; Robb, M. A.; Cheeseman, J. R.; Scalmani, G.; Barone, V.; Petersson, G. A.; Nakatsuji, H.; Li, X.; Caricato, M.; Marenich, A. V.; Bloino, J.; Janesko, B. G.; Gomperts, R.; Mennucci, B.; Hratchian, H. P.; Ortiz, J. V.; Izmaylov, A. F.; Sonnenberg, J. L.; Williams-Young, D.; Ding, F.; Lipparini, F.; Egidi, F.; Goings, J.; Peng, B.; Petrone, A.; Henderson, T.; Ranasinghe, D.; Zakrzewski, V. G.; Gao, J.; Rega, N.; Zheng, G.; Liang, W.; Hada, M.; Ehara, M.; Toyota, K.; Fukuda, R.; Hasegawa, J.; Ishida, M.; Nakajima, T.; Honda, Y.; Kitao, O.; Nakai, H.; Vreven, T.; Throssell, K.; Montgomery, J. A., Jr.; Peralta, J. E.; Ogliaro, F.; Bearpark, M. J.; Heyd, J. J.; Brothers, E. N.; Kudin, K. N.; Staroverov, V. N.; Keith, T. A.; Kobayashi, R.; Normand, J.; Raghavachari, K.; Rendell, A. P.; Burant, J. C.; Iyengar, S. S.; Tomasi, J.; Cossi, M.; Millam, J. M.; Klene, M.; Adamo, C.; Cammi, R.; Ochterski, J. W.; Martin, R. L.; Morokuma, K.; Farkas, O.; Foresman, J. B.; Fox, D. J. Gaussian (GAUSSIAN 16), Gaussian, Inc., Wallingford CT, **2016**.
(44) Becke, A. D. Density-functional exchange-energy approximation with correct asymptotic behavior. *Phys. Rev. A* **1988**, *38*, 3098-3100.
(45) Lee, C.; Yang, W.; Parr, R. G. Development of the Colle-Salvetti correlation-energy formula into a functional of the electron density. *Phys. Rev. B* **1988**, *37*, 785-789.
(46) Caldeweyher, E.; Ehlert, S.; Hansen, A.; Neugebauer, H.; Spicher, S.; Bannwarth, C.; Grimme, S. A generally applicable atomic-charge dependent London dispersion correction. *J. Chem. Phys.* **2019**, *150*, 154122.
(47) Grimme, S.; Antony, J.; Ehrlich, S.; Krieg, H. A consistent and accurate *ab initio* parametrization of density functional dispersion correction (DFT-D) for the 94 elements H-Pu. *J. Chem. Phys.* **2010**, *132*, 154104.
(48) Grimme, S.; Ehrlich, S.; Goerigk, L. Effect of the damping function in dispersion corrected density functional theory. *J. Comput. Chem.* **2011**, *32*, 1456-1465.
(49) Adamo, C.; Barone, V. Toward reliable density functional methods without adjustable parameters: The PBE0 model. *J. Chem. Phys.* **1999**, *110*, 6158-6170.
(50) Zhao, Y.; Truhlar, D. G. The M06 suite of density functionals for main group thermochemistry, thermochemical kinetics, noncovalent interactions, excited states, and transition elements: two new functionals and systematic testing of four M06-class functionals and 12 other functionals. *Theor. Chem. Accounts* **2008**, *120*, 215-241.
(51) Weigend, F.; Ahlrichs, R. Balanced basis sets of split valence, triple zeta valence and quadruple zeta valence quality for H to Rn: Design and assessment of accuracy. *Phys. Chem. Chem. Phys.* **2005**, *7*, 3297-3305.
(52) Rappoport, D.; Furche, F. Property-optimized Gaussian basis sets for molecular response calculations. *J. Chem. Phys.* **2010**, *133*, 134105.
(53) Dovesi, R.; Erba, A.; Orlando, R.; Zicovich-Wilson, C. M.; Civalleri, B.; Maschio, L.; Rérat, M.; Casassa, S.; Baima, J.; Salustro, S. Kirtman, B. Quantum-mechanical condensed matter simulations with CRYSTAL. *WIREs Comput. Mol. Sci.* **2018**, *8*, e1360.
(54) Dunning, T. H. Gaussian basis sets for use in correlated molecular calculations. I. The atoms boron through neon and hydrogen. *J. Chem. Phys.* **1989**, *90*, 1007-1023.
(55) Bergner, A.; Dolg, M.; Küchle, W.; Stoll, H.; Preuß, H. *Ab initio* energy-adjusted pseudopotentials for elements of groups 13–17. *Mol. Phys.* **1993**, *80*, 1431-1441.
(56) Martin, J. M. L.; Sundermann, A. Correlation consistent valence basis sets for use with the Stuttgart–Dresden–Bonn relativistic effective core potentials: The atoms Ga–Kr and In–Xe. *J. Chem. Phys.* **2001**, *114*, 3408-3420.
(57) Steenbergen, K. G.; Gaston, N.; Müller, C.; Paulus, B. Method of increments for the halogen molecular crystals: Cl, Br, and I. *J. Chem. Phys.* **2014**, *141*, 124707.
(58) Humphrey, W.; Dalke, A.; Schulten, K. VMD: Visual molecular dynamics. *J. Mol. Graph.* **1996**, *14*, 33-38.
(59) Lu, T.; Chen, F. Multiwfn: A multifunctional wavefunction analyzer. *J. Comput. Chem.* **2012**, *33*, 580-592.
(60) Lu, T. A comprehensive electron wavefunction analysis toolbox for chemists, Multiwfn. *J. Chem. Phys.* **2024**, *161*, 082503.
(61) Zhang, J.; Lu, T. Efficient evaluation of electrostatic potential with computerized optimized code. *Phys. Chem. Chem. Phys.* **2021**, *23*, 20323-20328.
(62) Lu, T.; Chen, F. Atomic dipole moment corrected Hirshfeld population method. *J. Theor. Comput. Chem.* **2012**, *11*, 163-183.
(63) Mahjoub, A. R.; Seppelt, K. The Structure of IF_6^-. *Angew. Chem. Int. Ed.* **1991**, *30*, 323-324.
